# Integrative single-cell and spatial transcriptomics uncover ELK4-mediated mechanisms in *NDUFAB1*+ tumor cells driving gastric cancer progression, metabolic reprogramming, and immune evasion

**DOI:** 10.3389/fimmu.2025.1591123

**Published:** 2025-07-04

**Authors:** Yuwei Sun, Wenyang Nie, Zhikai Xiahou, Xiaojing Wang, Wenjia Liu, Zongkai Liu, Zhiheng Lin, Zhaidong Liu

**Affiliations:** ^1^ College of First Clinical Medicine, Shandong University of Traditional Chinese Medicine, Jinan, China; ^2^ China Institute of Sport and Health Science, Beijing Sport University, Beijing, China; ^3^ Department of Rheumatology and Immunology, Tongren Hospital, School of Medicine, Shanghai Jiao Tong University, Shanghai, China; ^4^ Department of Oncology, Affiliated Hospital of Shandong University of Traditional Chinese Medicine, Jinan, China; ^5^ Department of Gynecology, Longhua Hospital, Shanghai University of Traditional Chinese Medicine, Shanghai, China

**Keywords:** gastric cancer, scRNA-seq, spatial transcriptomics, tumor microenvironment, metabolic reprogramming, immune evasion

## Abstract

**Background:**

Globally, gastric cancer (GC) stands as the fifth most prevalent form of malignant neoplasm and represents a significant contributor to mortality associated with oncological conditions. Despite advancements in therapeutic strategies for GC, the outcomes for patients with advanced stages of the disease continue to be unfavorable, largely due to tumor heterogeneity and the challenges posed by resistance to therapeutic agents. Metabolic reprogramming is pivotal in driving the advancement of GC, contributing to the development of resistance to pharmacological treatments and facilitating the cancer’s ability to evade immune surveillance. Developing multi-target comprehensive treatment strategies by integrating tumor microenvironment (TME) modulation holds promise for significantly improving therapeutic efficacy.

**Methods:**

The study analyzed GC and identified key cell subtypes by integrating data derived from single-cell RNA-sequencing (scRNA-seq) alongside spatial transcriptomics information. Cell type identification was accomplished using the tool of Seurat, and the spatial distribution of cell types was revealed through the Robust Cell Type Decomposition technique. CellChat was used to analyze the interactions between key cell subtypes and other cells, and the “StLearn” package was employed to investigate spatial cell communication in depth. Additionally, the functional role of the key molecule ELK4 was validated through *in vitro* experiments.

**Results:**

This research utilized scRNA-seq combined with spatial transcriptomics to comprehensively analyze GC, identifying the C1 *NDUFAB1*+ subtype, which exhibited high proliferative activity, metabolic reprogramming capabilities, and immune evasion properties. It was found that the C1 *NDUFAB1*+ subtype closely interacted with fibroblasts and pericytes via the PARs signaling pathway. Additionally, *in vitro* experiments confirmed that knockdown of ELK4 substantially curbed tumor cell proliferation, migration, and invasion.

**Conclusion:**

This study revealed the main significance of the C1 *NDUFAB1*+ subtype in GC, elucidating its core mechanisms in tumor progression, metabolic reprogramming, and immune evasion. ELK4 was identified as a key regulatory factor that markedly enhanced the proliferation, migratory capacity, and invasive potential of tumor cells, while changes in the TME were a driving force behind immune suppression and drug resistance. The findings underscored the importance of developing specific therapeutic targets, targeting metabolic reprogramming, and overcoming immune evasion, providing new theoretical foundations.

## Introduction

Gastric cancer (GC) is positioned as the fifth leading frequent type of cancer globally and holds a significant position among cancer-related causes of death, representing a significant risk to public health (1, 2). Referring to the global cancer data from 2022, the incidence of GC surpassed 968,000, with nearly 660,000 deaths, representing roughly 6.8% of total cancer mortality (3). The incidence of GC exhibits notable regional variations, with East Asia being a high-incidence region, while the African continent reports relatively lower rates (1, 3). Based on histological characteristics, clinical behavior, and molecular features, GC can be classified into several distinct types, with intestinal type and diffuse type being the two primary histological categories (4, 5). The intestinal type is frequently linked to intestinal metaplasia and Helicobacter pylori infection, showing higher prevalence in regions with elevated GC rates. On the other hand, the diffuse type is associated with worse clinical results and occurs frequently found in younger individuals and female patients (6, 7).

GC treatment primarily includes surgical resection, radiotherapy, chemotherapy, immunotherapy, and targeted treatment (8). Surgical resection remains the primary treatment for early-stage GC, but is associated with high postoperative recurrence rates and significant risk of distant metastasis (9). For advanced-stage patients, while chemotherapy can prolong survival (10), it often leads to severe adverse effects including myelosuppression and neurotoxicity (11). In recent years, immune checkpoint blockers (like PD-1/PD-L1 blockers) have exhibited certain efficacy in some GC patients (8, 12), but the response rate remains low, and some patients develop resistance after treatment. Clinical data showed that the objective response rate of single-agent PD-1/PD-L1 inhibitors in advanced GC patients was approximately 9%-14% (13, 14). More importantly, some advanced GC patients developed resistance following immune checkpoint inhibitor treatment (15). Drug resistance may arise from various molecular mechanisms, such as abnormal activation of the epidermal growth factor receptor (EGFR) signaling cascade (16–18), dysregulation of the PI3K/AKT/mTOR pathway (19–21), and the role of immunosuppressive cells in the tumor microenvironment (TME) (22). Additionally, research has demonstrated that alterations in cellular metabolism greatly aid in tumor advancement and resistance to therapeutic agents, and immune evasion of GC (23). GC cells undergo metabolic reprogramming, such as enhanced glycolysis, glutamine metabolism, and fatty acid synthesis, to fulfill the necessary energy for rapid cell division and maintain survival advantages (24, 25). Therefore, combining research on metabolic reprogramming with TME modulation strategies to develop multi-target, multi-level comprehensive treatment approaches hold promise for significantly boosting the efficacy of GC therapy and providing new breakthroughs to overcome drug resistance and tumor heterogeneity.

The recent innovations of single-cell RNA-sequencing (scRNA-seq) platform has offered superior precision in the study of GC (26–29). This technology helps identify cell subtypes and metabolic features associated with drug resistance (30–32), thereby guiding the combination of targeted drugs and immune checkpoint inhibitors. It can also be used to monitor dynamic changes in tumor cells and the TME during treatment, providing real-time evidence for adjusting treatment plans. Furthermore, the introduction of spatial transcriptomics (ST) has further expanded the depth of research, with its advantage lying in the ability to resolve the spatial distribution and interactions of different cell types within the TME, offering a more comprehensive perspective on tumor heterogeneity and TME complexity.

In this study, we incorporated ST following the implementation of scRNA-seq to thoroughly investigate tumor heterogeneity and spatial distribution characteristics within the GC TME. Through scRNA-seq, we identified four tumor cell subtypes, among which the C1 *NDUFAB1*+ subtype exhibited significant metabolic activity and stem cell-like properties. Its metabolic features were closely associated with metabolic reprogramming, suggesting that this subtype may play a key driving role in tumor progression. Additionally, we discovered strong interactions between C1 *NDUFAB1*+ subtype and fibroblasts as well as pericytes throughout the TME, primarily mediated through the PARs signaling pathway, particularly via the PRSS3-F2R ligand-receptor pair. ST further confirmed the spatial enrichment of C1 *NDUFAB1*+ subtype in specific regions of GC tissues, underlining their major contribution to tumor development. To elucidate further the molecular regulatory mechanisms of C1 *NDUFAB1*+ subtype, we identified ELK4, a key transcription factor (TF) highly expressed in this subtype. ELK4 has been recognized as a promising target for treating multiple malignancies (33, 34), as inhibiting its expression or activity can constrain the proliferation, migration, and immune evasion of tumor cells, while enhancing sensitivity to chemotherapy and immunotherapy. To validate the regulatory capacity of ELK4 in GC, we executed *in vitro* tests with two GC cell lines (NCI-N87 and AGS). The findings indicated that silencing ELK4 significantly impaired the proliferation, migration, and invasion capabilities of GC cells. Furthermore, based on genes associated with C1 *NDUFAB1*+ subtype, we developed and verified a predictive risk scoring model, revealing that individuals in the high-risk group exhibited poorer clinical outcomes, and their TME exhibited significant immunosuppressive characteristics, further supporting the significant impact of C1 *NDUFAB1*+ subtype in tumor progression and immune evasion.

Our study comprehensively revealed the key role of C1 *NDUFAB1*+ subtype in metabolic reprogramming, tumor progression, and immune microenvironment regulation in GC. We identified the C1 *NDUFAB1*+ subtype with distinct metabolic characteristics using scRNA-seq combined with ST, and investigated its unique spatial distribution patterns and interaction networks with stromal cells in the TME. Furthermore, we successfully established a risk scoring model based on the C1 *NDUFAB1*+ subtype. These discoveries open new avenues for the development of combined therapies targeting immune evasion and metabolic regulation, while also offering new theoretical foundations and potential targets for the precision treatment of GC.

## Materials and methods

### Acquisition of GC data

In this study aimed at scRNA-seq analysis of GC, we accessed the Gene Expression Omnibus (GEO) database (https://www.ncbi.nlm.nih.gov/geo/) using the accession number GSE183904. Subsequently, we selected 20 GC tissue samples for analysis. Additionally, we obtained bulk RNA-seq data from The Cancer Genome Atlas (TCGA) website (https://portal.gdc.cancer.gov/). As the datasets utilized in this research were sourced from publicly available repositories, no ethical review was required.

### Single-cell sequencing data handling and interpretation

During the data import phase, we used R software (v4.2.2) and the Seurat package (v4.3.0) to perform initial handling of the single-cell sequencing data (35, 36). To ensure data reliability, we excluded low-quality cells by purifying, filtering, and removing doublets (37–39). The filtering criteria included (1): nFeature range (300–5,000) (2); nCount range (500–50,000) (3); mitochondrial gene expression proportion (≤25%) (4); red blood cell gene expression proportion (≤5%). After strict filtering, a total of 92,566 high-quality cells were preserved for subsequent study.

Data normalization was a critical step in the analysis. We used the “NormalizeData” function (40–42) to normalize the samples. To capture heterogeneity among cells, we employed the “FindVariableFeatures” function (43–45) to identify the top 2,000 most variable genes (46–48). Subsequently, we standardized the gene expression data using the “ScaleData” function (49–51) and performed Principal Component Analysis (PCA) (41, 52, 53). Batch effects were corrected using the Harmony R package (v0.1.1). Ultimately, dimensionality reduction and visualization were achieved by applying Uniform Manifold Approximation and Projection (UMAP) based on the top 30 principal components, further revealing differences in gene expression among cells (54–56).

### Identification and annotation of cell types

We utilized the “FindClusters” and “FindNeighbors” functions (57) to perform clustering analysis on the cells, which initially determined the distribution of cell subtypes. To further dissect the heterogeneity of GC cells, we employed the “FindAllMarkers” function to detect genes with differential expression patterns within each subtype and annotated the cell subtypes by calculating the average expression of marker genes. The selection of marker genes integrated known information from the CellMarker database (http://xteam.xbio.top/CellMarker/) and relevant data from the literature, with manual curation ensuring the accuracy of the annotations.

### Copy number variation assessment

We employed the inferCNV R program (v1.6.0) (58) to analyze scRNA-seq data, distinguishing malignant tumor cells from normal cells by calculating CNV levels. Using endothelial cells (ECs) as a reference, we effectively identified chromosomal CNVs in tumor cells. By comparing gene expression intensities between tumor cells and normal cells, inferCNV inferred amplifications or deletions in chromosomal regions.

### Analysis of spatial transcriptome

We collected ST 1 and ST 2 slides from the ST dataset (GSE251950) and employed the Robust Cell Type Decomposition (RCTD) technique to map cell types identified in the scRNA-seq dataset to the spatial ST data, aiming to reveal the distribution of different cell types across spatial regions. First, we used the “FindAllMarkers” function to identify marker genes for each cell type, filtering for markers with a positive log2-transformed fold change. Subsequently, following the standard RCTD analysis pipeline, we analyzed the reference scRNA-seq data and Visium ST data in a complete doublet mode. To further determine the spatial distribution of cell types, we localized the cell types identified in the scRNA-seq data to spatial regions significantly enriched by Multimodal Intersection Analysis (MIA). Additionally, we utilized the “StLearn” package in Python to explore interactions in detail within spatial regions.

### Biological function enrichment analysis

Using the “ClusterProfiler” package, we executed functional profiling on differentially expressed genes (DEGs) in tumor cell subtypes through Gene Ontology (GO) (59–62) and Kyoto Encyclopedia of Genes and Genomes (KEGG) pathway enrichment (63–65). Besides, Gene Set Enrichment Analysis (GSEA) was employed to evaluate the overall expression patterns of gene sets, further revealing the functional characteristics of tumor cell subtypes. To more comprehensively analyze the variability in gene expression data, we applied Gene Set Variation Analysis (GSVA), computing the enrichment scores of specific gene sets across individual samples.

### Differentiation trajectory and stemness analysis

Based on the Slingshot (v2.6.0) trajectory inference framework, we constructed a developmental trajectory map of tumor cell subtypes and revealed the temporal characteristics of their differentiation pathways through pseudotime analysis. Smooth trajectory curves were derived using the “getLineages” and “getCurves” tools, and changes in cell expression levels were visualized in reduced-dimensional space. The initial and final points of the differentiation trajectories were determined based on the biological characteristics of GC tumor cell subtypes and the expression patterns of their marker genes. Additionally, we employed CytoTRACE to evaluate the stemness levels of different tumor cell subtypes, with scores ranging from 0 to 1, where higher scores indicated stronger cell stemness.

### Cell communication

We leveraged the “CellChat” (v1.6.1) (66) for cell type interaction analysis in the GC microenvironment, constructing a cell communication network. The “netVisual_diffInteraction” function was used to visualize the changes in communication intensity between cells, while the “identifyCommunicationPatterns” function was employed to identify complex communication patterns. By integrating the CellChatDB database, we identified key signaling pathways and ligand-receptor pairs. A *P*-value cutoff of 0.05 was established in the analysis to assess the statistical significance of cell-cell interactions.

### Transcriptional regulatory network analysis

We used the pySCENIC package (v0.10.0) (67) to reconstruct the gene regulatory network of tumor cells in GC, identifying stable cell states and assessing transcriptional activity. AUCell (68) was employed to compute the activity of regulons, with a score threshold set at 0.2, and significance was evaluated through 100 random permutations. Key TFs were selected based on their regulon specificity scores and AUCell activity.

### Construction and validation of a prognostic model for GC

First, we extracted pertinent data from the TCGA database and identified eight genes with significant prognostic relevance through univariate Cox regression analysis (69–72). To mitigate multicollinearity among the genes, LASSO (73, 74) and multivariate Cox regression analysis was subsequently employed, ultimately identifying seven key prognostic genes and calculating their coefficient values. A risk scoring model was developed using the following formula: Risk Score = $\sum_{\text{i}}^{\text{n}}\text{Xi }\times \text{ Yi}$ , with X denoting the coefficient and Y denoting the gene expression. Using the optimal threshold identified by the “surv_cutpoint” algorithm, patients were separated into high- and low-risk groups. To validate the predictive ability of the model, we utilized Kaplan-Meier and Receiver Operating Characteristic (ROC) curves to assess the model’s predictive accuracy (75–79). Additionally, we constructed a nomogram incorporating age, gender, race, stage, risk score, and TNM classification (80). The model was further validated through Area Under the Curve (AUC) values (81–83) based on both the C-index and true positive rate.

### Analysis of the immune landscape and therapeutic response

The CIBERSORT and ESTIMATE (84, 85) were used to assess immune infiltration. Subsequently, we utilized the Tumor Immune Dysfunction and Exclusion (TIDE) program to evaluate the response to immunotherapy in the high- and low-risk groups. Afterward, we employed the “pRRophetic” package (v0.5) to estimate the half-maximal inhibitory concentration (IC50) values for drug.

### Cell culture

The NCI-N87 GC cell line was cultured in 1640 basal medium enriched with 10% fetal bovine serum (FBS), 1% penicillin-streptomycin (P/S), 1% GlutaMAX-1 glutamine, and 1% sodium pyruvate, while the AGS GC cell line was cultured in F12K medium supplemented with 10% premium FBS and 1% penicillin-streptomycin (P/S). Both GC cell lines were maintained under standard conditions (37°C, 5% CO_2_, 95% humidity).

### Cell transfection

In the cell transfection experiment, we utilized small interfering RNAs (siRNAs) provided by GenePharma (Suzhou, China) to inhibit ELK4 expression. Cells were grown in 6-well plates until achieving 50% confluency, followed by transfection using Lipofectamine 3000 RNAiMAX (Invitrogen, USA). Two specific siRNA sequences, siELK4-1 (CAUUCAACAUGAUUGCAUU) and siELK4-2 (CUCAGAUACUAUUAUGUAA), were designed for the experiment, along with a negative control siRNA (si-NC) as a control.

### Cell viability assay

We employed the CCK-8 method to evaluate the viability of transfected NCI-N87 and AGS cells. Cells were plated in 96-well plates at a concentration of 5×10³ cells per well and incubated for 24 h to allow proper adherence. Subsequently, 10 μL of CCK-8 reagent (A311-01, Vazyme) was added to each well, gently mixed, and incubated at 37°C in the dark for 2 h. Following incubation, absorbance readings were taken at 450 nm using a microplate spectrophotometer (A33978, Thermo). The experiment was conducted daily from day 1 to day 4 post-transfection, and the absorbance values were recorded and averaged. A curve depicting changes in cell viability over time was plotted to visually reflect the dynamic changes in cell viability levels.

### Quantitative real-time polymerase chain reaction

We first extracted RNA from the cells using TRIzol reagent, followed by reverse transcription. Then, the amplification process was monitored in real-time using fluorescent dyes. The primer sequences used in the experiment were: F: GGATTCGCAAGAACAAGCCT, R: TCAATCCTGCCCACTGTCAT.

### 5-ethynyl-2’-deoxyuridine proliferation assay

We seeded the transfected NCI-N87 and AGS cells in 6-well plates at a density of 5×10³ cells per well and cultured them for 24 h to allow proper adherence. Subsequently, 2× EDU working solution was added to the medium, and the cells were incubated at 37°C for 2 h. Post-incubation, the supernatant was aspirated, and the adherent cells were subjected to dual PBS washes for the removal of non-incorporated reagent. Following this step, the cellular specimens underwent fixation in 4% paraformaldehyde for a duration of 0.5 h. Afterward, a permeabilization procedure was performed with a mixture of 2 mg/mL glycine and 0.5% Triton X-100 to optimize staining performance.

To conclude the procedure, the cells were treated with a 1:1 mixture of 1ml 1X Apollo and Hoechst 33342 solution for 0.5 h to label proliferating cells and nuclei. After staining, images were observed and captured, and the frequency of EDU-incorporated cells was quantified and statistically evaluated.

### Wound healing assay

Transfected cellular populations were initially seeded into 6-well tissue culture plates and allowed them to grow until they achieved nearly 95% confluency. Uniform linear wounds were generated using a sterile 200 μL pipette tip on the cell monolayer to simulate wounds. Post-scratching, the wells underwent additional washing steps, and serum-free medium was applied to prevent serum interference with cell migration. Following this, images of the wound areas were then acquired at the initial time point (0 h) and after 48 h to track the migratory behavior of the cells over time. After the experiment, the scratch widths were quantitatively analyzed using Image-J software to calculate the cell migration rate.

### Transwell assay

After a 1-day serum starvation period, Matrigel (BD Biosciences, USA) was thoroughly mixed with the cell suspension and applied to the upper compartment of the transwell, with serum-added medium being introduced into the lower chamber. Upon completion of a 2-day incubation in culture dishes, cellular specimens were fixed using 4% paraformaldehyde. Finally, crystal violet staining was applied to quantitatively assess migratory and invasive capabilities.

### Statistical analysis

The research employed R and Python computational tools for statistical computations and data interpretation, with the Wilcoxon rank-sum test and Spearman correlation methodology being the principal approaches for evaluating intergroup statistical significance. For the significance determination criteria, a two-tailed test was used to calculate *P*-values, with a statistical threshold set at 0.05. The statistical significance levels were denoted using asterisks, with * representing *P*-values below 0.05, ** indicating *P* < 0.01, *** signifying *P* < 0.001, and **** marking *P*-values less than 0.0001. This hierarchical system enhanced the interpretability of the results by quantifying the strength of differences.

## Results

### Single-cell profiling uncovered heterogeneity and subtype-specific molecular signatures in GC

**Figure 1:**
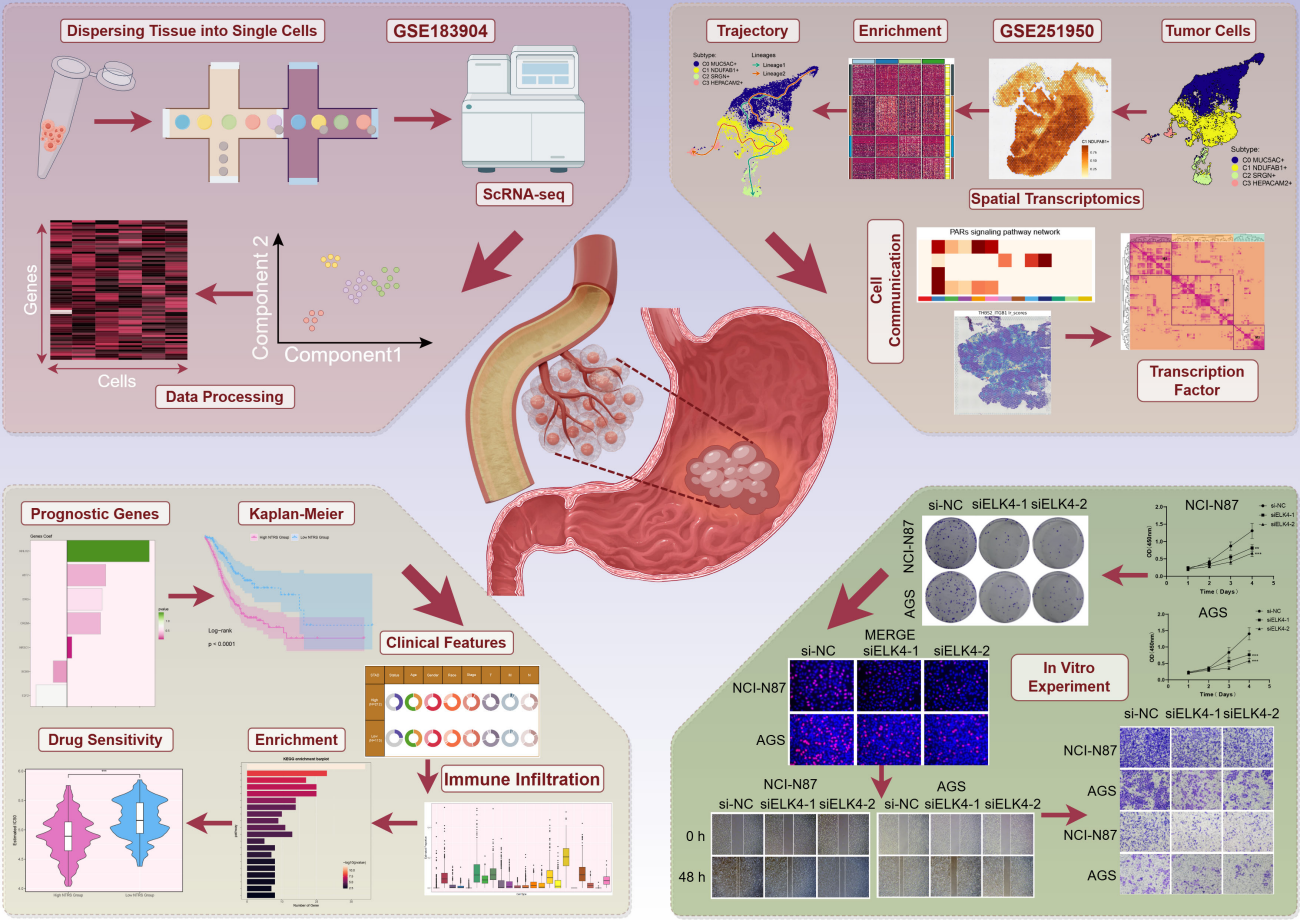
GC scRNA-seq: analysis workflow. The analysis process of GC scRNA-seq data covered data normalization, identification of key cell subtypes, and functional interpretation.

**Figure 2:**
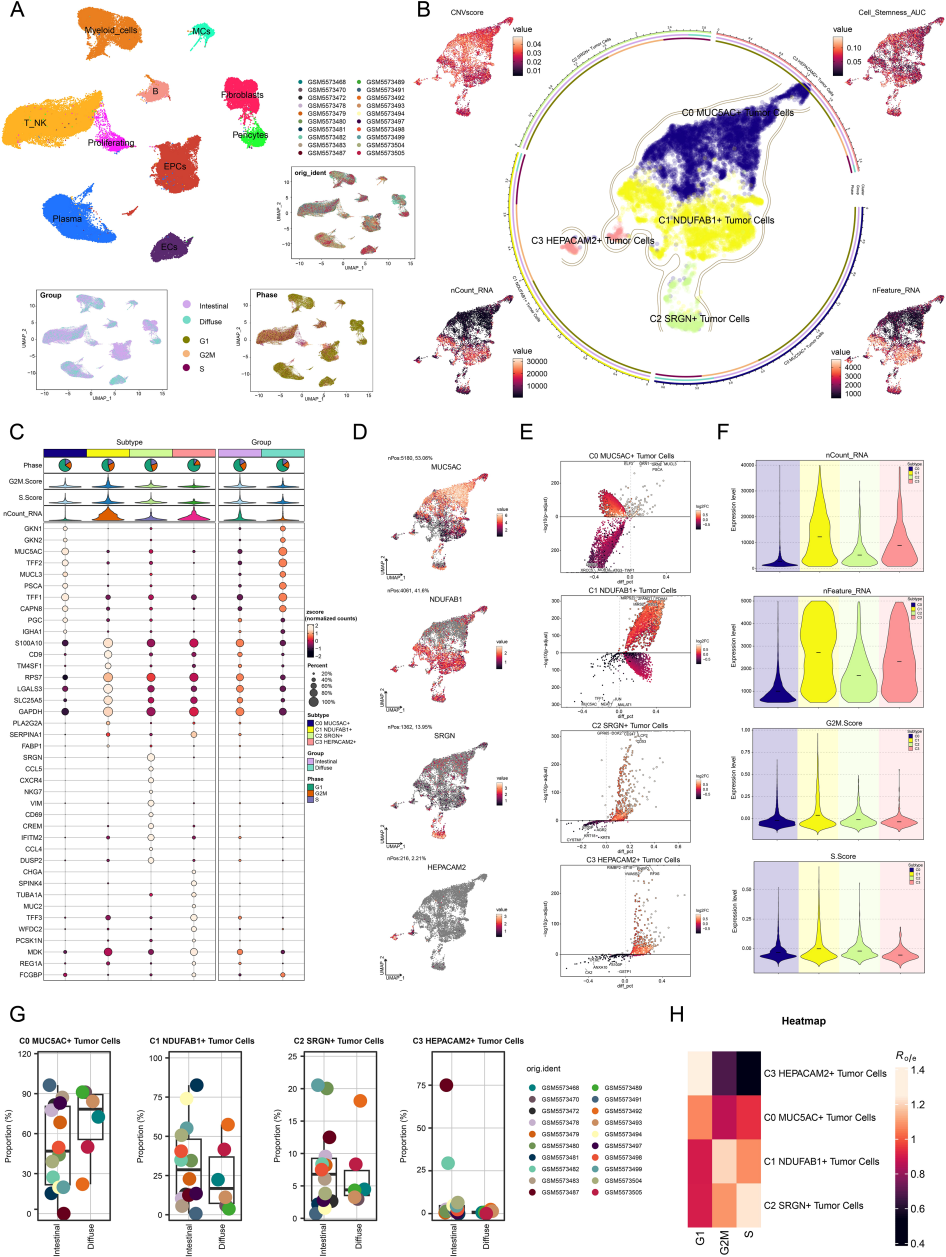
Detailed single-cell profiling of GC. **(A)** UMAP plot in the upper left corner displayed the distribution of 10 different cell types, while the UMAP plots in the lower right corner displayed the distribution of 20 different samples, cell cycle phases, and groups, respectively. **(B)** The circular plot showed the clustering of four tumor cell subtypes identified in GC, outlined by contour curves. The outer, middle, and inner axes represented the logarithmic scale of the clusters for each tumor cell subtype, along with group and phase. UMAP plots were arranged in a clockwise direction starting from the top left corner, displaying the expression distributions of CNVscore, Cell-Stemness-AUC, nFeature-RNA, and nCount-RNA across all tumor cells. **(C)** Bubble plot displayed the differential expression of the top 10 marker genes across the four tumor cell subtypes and two groups. The pie charts displayed the proportions of G1, G2/M, and S phases, while the violin plots presented the expression levels of G2/M.Score, S.Score, and nCount-RNA. The size of the bubbles indicated the percentage of gene expression, and the color intensity represented the level of gene expression. **(D)** UMAP plots showcased the distribution of named genes for each tumor cell subtype. **(E)** Volcano plots highlighted the differentially upregulated and downregulated genes in each tumor cell subtype. **(F)** Violin plots illustrated the expression levels of nCount-RNA, nFeature-RNA, G2/M.Score, and S.Score across different tumor cell subtypes. **(G)** Box plots described the proportion of different samples in each subtype across the two groups. **(H)** Heatmap assessed phase preference for each subtype using the Ro/e score.

Based on GC scRNA-seq data, we thoroughly analyzed the cellular composition and functional characteristics of its microenvironment. Our analysis workflow was shown in **Figure 1**. We first performed quality control and batch effect correction on the collected GC tissues. Then, after dimensionality reduction and clustering of the high-quality filtered cells, we identified 10 major cell types, including epithelial cells (EPCs), ECs, fibroblasts, myeloid cells, pericytes, mast cells (MCs), T cells and NK cells, B cells, plasma cells, and proliferating cells. The distribution profiles of 20 individual samples were additionally presented in **Figure 2A**, stratified by cell cycle phases (G1, G2/M, S) and group types (intestinal, diffuse). The heterogeneity of EPCs in the TME is a complex and significant phenomenon, profoundly influencing tumor development and treatment response. GC, a cancerous growth arising from the stomach mucosal epithelium, is closely associated with EPCs in its occurrence and progression (86). For the discrimination of cancerous cells from normal cellular populations and examination of tumor heterogeneity, we used ECs as a comparative baseline and identified tumor cells within EPCs through inferCNV analysis (**Supplementary Figure 1**). Following this, we performed a more detailed examination regarding the tumor cells and classified four tumor cell subtypes based on the expression levels of marker genes (C0 *MUC5AC*+ subtype, C1 *NDUFAB1*+ subtype, C2 *SRGN*+ subtype, C3 *HEPACAM2*+ subtype). In **Figure 2B**, we demonstrated the clustering of these four tumor cell subtypes and the expression distribution of CNVscore, Cell-Stemness-AUC, nFeature-RNA, and nCount-RNA across all tumor cells. Additionally, we displayed the expression of the top 10 marker genes, the distribution of named genes, as well as the significantly upregulated and downregulated genes in each subtype (**Figures 2C–E**). Interestingly, we observed that C1 *NDUFAB1*+ subtype exhibited higher expression levels of nCount-RNA, nFeature-RNA, G2/M.Score, and S.Score in **Figure 2F**. Therefore, we speculated that C1 *NDUFAB1*+ subtype might be in a more active cell cycle state and could potentially possess stronger malignant characteristics. In **Figure 2G**, we depicted the proportion of different samples for each subtype in the two groups, finding that the percentage of intestinal samples in C1 *NDUFAB1*+ subtype was higher than that of diffuse samples. As expected, the cell cycle phases of C1 *NDUFAB1*+ subtype showed a preference for the G2/M and S phases (**Figure 2H**).

### Spatial multi-omics features and co-localization of key subtypes in GC

**Figure 3:**
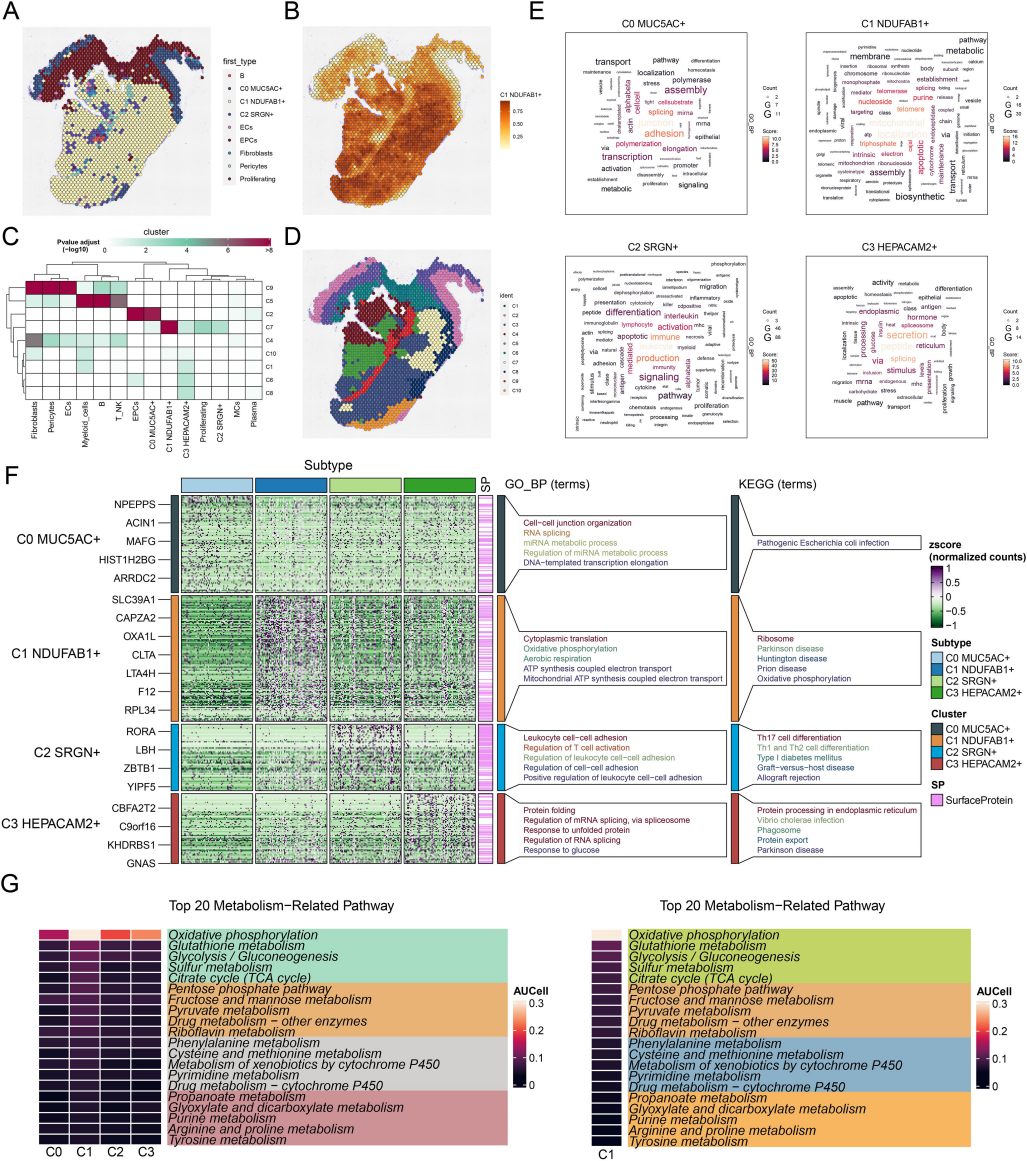
Spatial distribution features and enrichment analysis of tumor cell subtypes in GC. **(A)** The ST feature map demonstrated the first cell types inferred at selected points on ST 1 slide. Each point represented the cell type with the highest probability within that location. **(B)** The ST feature map revealed the spatial expression pattern of the C1 *NDUFAB1*+ subtype. The intensity of the color indicated the relative strength of expression. **(C)** MIA analysis assessed the enrichment degree of spatial clusters associated with various cell types on ST 1 slide. **(D)** The results of spatial spot clustering on ST 1 slide were visualized. **(E)** The word cloud diagrams depicted the main biological processes of each tumor cell subtype. **(F)** Heatmap illustrated the top 5 enriched GOBP and KEGG terms in tumor cell subtype. **(G)** Heatmaps respectively showed the top 20 enriched metabolism-related pathways across all tumor cell subtypes and C1 *NDUFAB1*+ subtype.

To investigate the spatial multi-omics characteristics of GC and explore the spatial expression patterns of key molecular events during tumor progression, we conducted ST analysis on two collected GC tissue sections. We utilized the RCTD deconvolution method to display the first cell types inferred at selected points on ST 1 slide in **Figure 3A**. **Figure 3B** illustrated the ST landscape of C1 *NDUFAB1*+ subtype, which was consistent with the first cell types inferred above. To validate the accuracy of RCTD, we further analyzed the data using the MIA method (**Figures 3C, D**). Analysis revealed that the C7 cluster showed maximal enrichment within C1 *NDUFAB1*+ subtype, and the spatial distribution characteristics of the C7 cluster on ST 1 slide were similar to those of C1 *NDUFAB1*+ subtype. In addition, we performed RCTD analysis on ST 2 slide, which revealed high expression in regions spatially corresponding to C1 *NDUFAB1*+ subtype (**Supplementary Figures 2A, B**). Furthermore, **Supplementary Figure 2C** demonstrated that nCount-Spatial, nFeature-Spatial, G2/M.Score, and S.Score exhibited spatial expression patterns similar to those of C1 *NDUFAB1*+ subtype.

### Biological functions and metabolic analysis of tumor cell subtypes in GC

These characteristics of C1 *NDUFAB1*+ subtype suggested that they might be a key driver subtype in tumor progression. Consequently, we proceeded to investigate their biological functions. **Figures 3E, F** illustrated the main biological processes enriched by DEGs in different tumor cell subtypes. We found that C0 *MUC5AC*+ subtype was primarily associated with junction, adhesion, and splicing, and were mainly enriched in pathways such as cell-cell junction organization, RNA splicing, miRNA metabolic process, regulation of miRNA metabolic process, and DNA-templated transcription elongation. C1 *NDUFAB1*+ subtype was mainly associated with mitochondrial, localization, and triphosphate, and were predominantly concentrated in pathways such as cytoplasmic translation, oxidative phosphorylation, aerobic respiration, ATP synthesis coupled electron transport, and mitochondrial ATP synthesis coupled electron transport. C2 *SRGN*+ subtype was primarily associated with leukocyte, production, and immune, and were mainly enriched in pathways such as leukocyte cell-cell adhesion, regulation of T cell activation, regulation of leukocyte cell-cell adhesion, regulation of cell-cell adhesion, and positive regulation of leukocyte cell-cell adhesion. C3 *HEPACAM2*+ subtype was mainly associated with peptide, secretion, and splicing, and demonstrating major representation in functional pathways such as protein folding, regulation of mRNA splicing and via spliceosome, response to unfolded protein, regulation of RNA splicing, and response to glucose. Meanwhile, in **Figure 3F**, we illustrated the KEGG terms enriched in each tumor cell subtype. C0 *MUC5AC*+ subtype was primarily associated with pathogenic Escherichia coli infection. C1 *NDUFAB1*+ subtype showed significant enrichment in ribosome, Parkinson disease, Huntington disease, prion disease, and oxidative phosphorylation. C2 *SRGN*+ subtype was predominantly linked to Th17 cell differentiation, Th1 and Th2 cell differentiation, type I diabetes mellitus, graft-versus-host disease, and allograft rejection. Finally, C3 *HEPACAM2*+ subtype was mainly related to protein processing in endoplasmic reticulum, vibrio cholerae infection, phagosome and protein export. Subsequently, in **Figure 3G**, we displayed the top 20 enriched metabolism-related pathways across different tumor cell subtypes. Notably, compared to other subtypes, the C1 *NDUFAB1*+ subtype showed predominant enrichment in oxidative phosphorylation, glutathione metabolism, glycolysis/gluconeogenesis, sulfur metabolism, and citrate cycle (TCA cycle). The above results indicated that C1 *NDUFAB1*+ subtype exhibited high metabolic activity, a characteristic that likely supported their rapid proliferation and survival, making them a key driver subtype in tumor progression. Meanwhile, C0 *MUC5AC*+ subtype, C2 *SRGN*+ subtype, and C3 *HEPACAM2*+ subtype played important roles in cell-cell connections, immune responses, and stress responses, respectively, highlighting the heterogeneity and complexity of the tumor.

### Differentiation trajectories and stemness analysis

**Figure 4:**
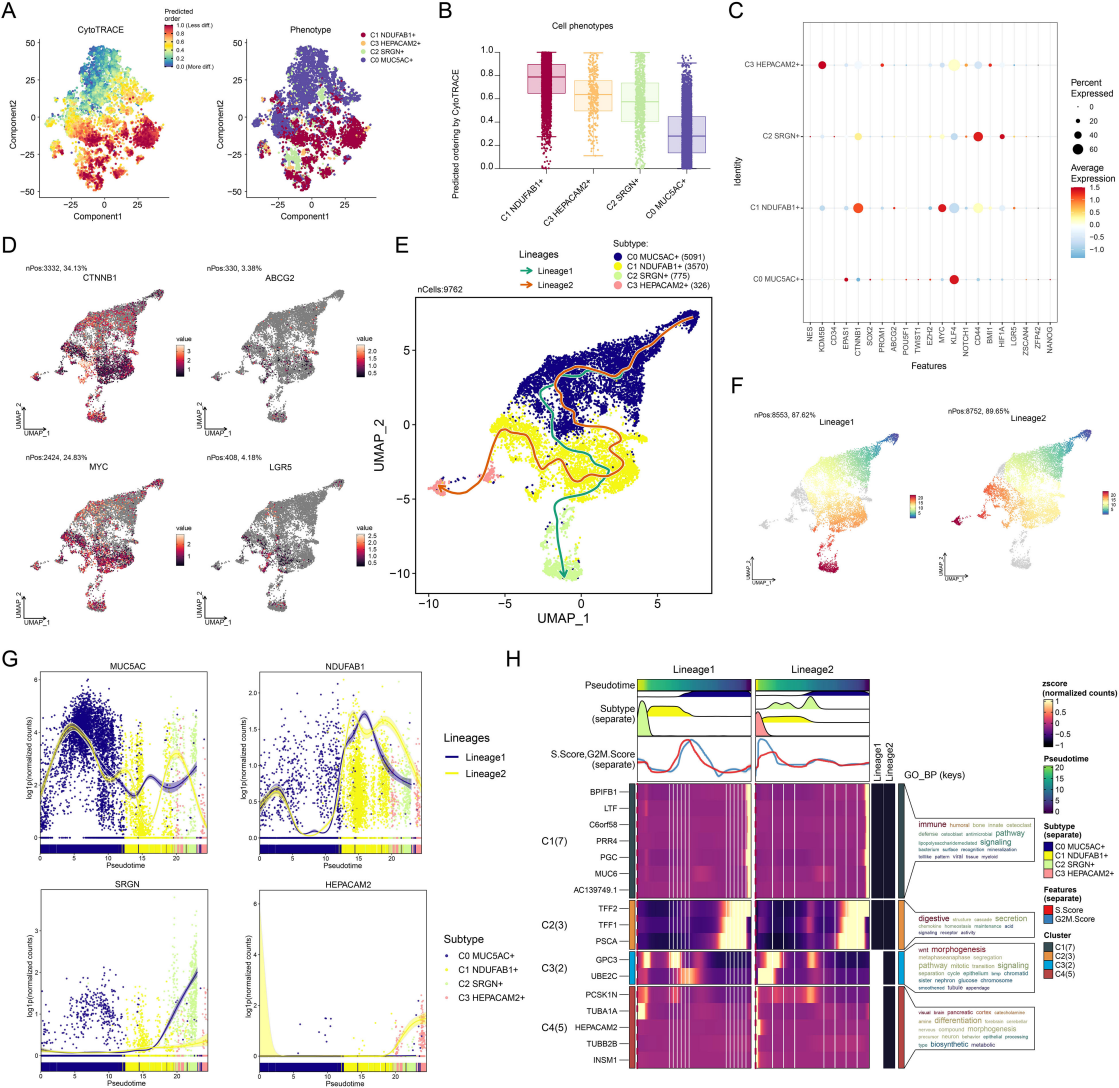
Developmental and differentiation features of tumor cell subtypes in GC. **(A)** The two-dimensional graphs depicted the CytoTRACE results of the predicted order (left) and distribution characteristics (right) for the four tumor cell subtypes. **(B)** Box plot described the stemness ranking of the tumor cell subtypes predicted by CytoTRACE. **(C)** Bubble plot displayed the differential expression of the top stemness genes for each tumor cell subtype. **(D)** UMAP plots visualized the distribution of significantly expressed stemness genes in C1 *NDUFAB1*+ subtype. **(E)** UMAP plot utilized Slingshot analysis to display two cellular differentiation lineages of the four tumor cell subtypes over time. Solid lines and arrows represented the differentiation trajectories and the direction of cell differentiation from naive to mature, respectively. **(F)** UMAP plots illustrated the distribution of lineage 1 and lineage 2 fitted by Slingshot, showing their progression along the inferred pseudotime order. **(G)** Dynamic trend plots depicted the pseudotime trajectories of named genes across four tumor cell subtypes in lineage 1 and lineage 2. **(H)** Heatmap demonstrated the key biological processes related to the two lineages in tumor cell differentiation, as presented in the GO enrichment analysis results. The ridge plots displayed the pseudotime density changes of the four tumor cell subtypes. The trajectory plots showed the pseudotime score changes of S.Score and G2/M.Score across the two lineages.

The differentiation process of tumor cells is one of the pivotal elements in tumorigenesis and progression, and stemness, as an important characteristic of tumor cells, directly influences their self-renewal, proliferation, and differentiation capabilities (87). Understanding the differentiation trajectories and stemness features of different tumor cell subtypes is crucial for revealing tumor heterogeneity, prognosis, and potential therapeutic targets. Therefore, we systematically explored the differentiation lineages and related characteristics of different tumor cell subtypes using a combination of analytical methods. First, we ranked the stemness of different tumor cell subtypes using the CytoTRACE method, and the observations supported that C1 *NDUFAB1*+ subtype possessed increased stemness properties, while C0 *MUC5AC*+ subtype exhibited lower stemness (**Figures 4A, B**). This finding suggested that C1 *NDUFAB1*+ subtype might exhibit enhanced capacity for self-renewal and differentiation. To further investigate their stemness features, we displayed the variations in the expression of stemness genes among distinct subtypes (**Figure 4C**) and visualized the distribution of significantly expressed stemness genes (*CTNNB1*, *ABCG2*, *MYC*, *LGR5*) in C1 *NDUFAB1*+ subtype (**Figure 4D**). The high expression patterns of these genes further supported the high stemness characteristics of C1 *NDUFAB1*+ subtype. To comprehensively understand the differentiation dynamics of tumor cells, we used Slingshot to evaluate the differentiation lineages of four subtypes (**Figure 4E**). The results showed that both lineage 1 and lineage 2 passed through C0 *MUC5AC*+ subtype in the early differentiation stage and diverged to different endpoints after passing through C1 *NDUFAB1*+ subtype. This observation indicated that C1 *NDUFAB1*+ subtype might serve as critical factors in cellular differentiation mechanisms. To further validate this hypothesis, we displayed the distribution of lineage 1 and lineage 2 fitted by Slingshot, clearly showing their progression along the inferred pseudotime order (**Figure 4F**). Additionally, the pseudotime trajectories of named genes in the four tumor cell subtypes within lineage 1 and lineage 2 were highly consistent with the Slingshot results (**Figure 4G**), further supporting our findings.

Based on these observations, we speculated that C1 *NDUFAB1*+ subtype might play a critical role in the differentiation process of GC cells. Specifically, C0 *MUC5AC*+ subtype exhibited lower stemness and higher differentiation levels, while C1 *NDUFAB1*+ subtype demonstrated higher stemness, proliferative capacity, and differentiation potential. To more deeply reveal the biological significance of these different lineages, we explored key biological processes associated with tumor cell differentiation (**Figure 4H**). The evidence suggested that C1 cluster was mainly tied to immune and humoral responses, C2 cluster was related to digestive and structural functions, and C3 cluster was closely linked to Wnt signaling pathway and morphogenesis. Additionally, C4 cluster was significantly associated with visual, brain, and pancreatic functions. These enrichment results not only revealed the potential functional differences among different tumor cell subtypes but also provided new insights into their roles in tumorigenesis and development.

### Cell-cell communication and signaling pathway regulatory network analysis

**Figure 5:**
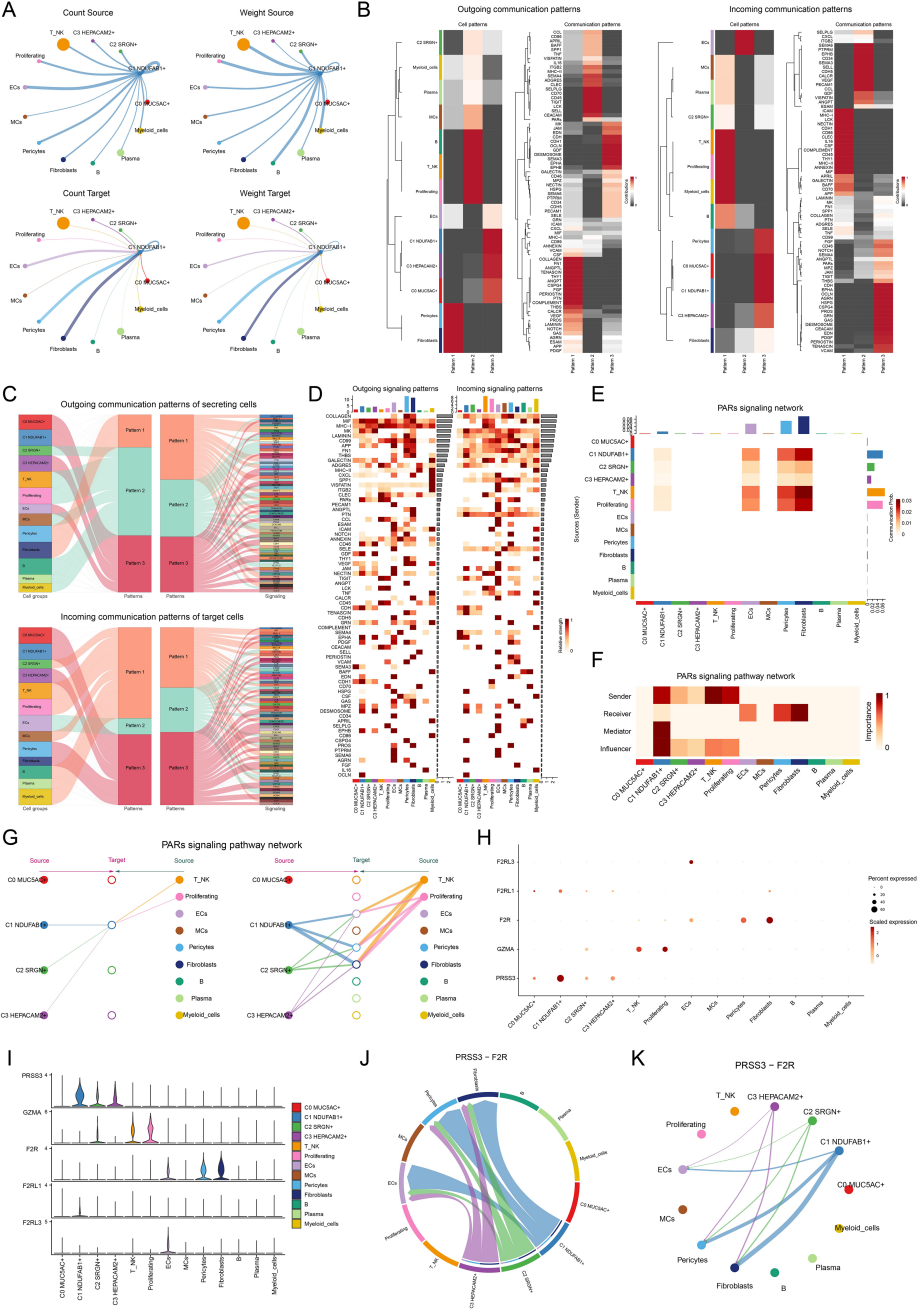
Cell communication in single-cell transcriptomics. **(A)** Circle diagrams represented the interactions between C1 *NDUFAB1*+ subtype as both the source (upper) and the target (lower) with other cell types in terms of the number (left) and intensity (right). **(B)** Heatmaps showed the outcoming (left) and ingoing (right) communication patterns of the four tumor cell subtypes and nine other cell types across three cell communication patterns, along with the contributions of different proteins in each communication pattern. **(C)** Sankey diagrams predicted the outgoing communication patterns (upper) of the four tumor cell subtypes and nine other cell types as secreting cells, and the incoming communication patterns (lower) as target cells, along with the signaling pathways under the three cell communication patterns. **(D)** Heatmaps displayed the relative intensity of various signaling pathways in the four tumor cell subtypes and nine other cell types under the outgoing and incoming signaling patterns, while the bar charts illustrated the relative signal intensity of different cell types. **(E)** Heatmap showed the communication probability of the four tumor cell subtypes and nine other cell types under the PARs signaling network. **(F)** Heatmap depicted the centrality scores within the PARs signaling pathway network. **(G)** Hierarchical diagram portrayed the interactions between four tumor cell subtypes and nine other cell types in the PARs signaling pathways network. **(H, I)** Bubble plot and violin plot compared the expression of significant ligands and receptors in the PARs signaling pathways across four tumor cell subtypes and nine other cell types. **(J, K)** Chord plot and circle diagram displayed the communication network in the PRSS3-F2R ligand-receptor pair.

To better elucidate the involvement of C1 *NDUFAB1*+ subtype in the TME, we investigated the complex interactions between tumor cells and other cell types. Using CellChat, we executed a detailed investigation of the communication networks between C1 *NDUFAB1*+ subtype as both source and target with other cell types (**Figure 5A**). We found that the interactions between C1 *NDUFAB1*+ subtype and fibroblasts, as well as pericytes, were stronger compared to other cell types. We examined the main driving patterns of all cell types and the interaction proteins under these patterns (**Figures 5B, C**). The investigations indicated that the outgoing and incoming signals of C1 *NDUFAB1*+ subtype were primarily driven by Pattern 3, involving significant contributions from CDH, EDN, OCLN, DESMOSOME, and EPHA. In **Figure 5D**, we further presented the relative signal strength of various signaling pathways across different cell types and all cell types under different signal patterns. Through signal network analysis, we discovered that C1 *NDUFAB1*+ subtype primarily communicated with fibroblasts and pericytes via the PARs signaling pathway (**Figure 5E**). Additionally, in **Figure 5F**, we observed that C1 *NDUFAB1*+ subtype mainly took on the responsibilities of sender, mediator, and influencer, while fibroblasts and pericytes acted as receivers. Subsequently, we used a hierarchical graph to reveal that C1 *NDUFAB1*+ subtype might regulate themselves through autocrine mechanisms, while influencing fibroblasts and pericytes through paracrine mechanisms (**Figure 5G**). Furthermore, we visualized the expression of key ligand-receptor pairs in the PARs signaling pathway across four tumor cell subtypes and nine other cell types (**Figures 5H, I**). We found that PRSS3 and F2R were highly expressed in C1 *NDUFAB1*+ subtype, fibroblasts, and pericytes. We then further analyzed the communication network within the PRSS3-F2R ligand-receptor pair, confirming that the interactions between C1 *NDUFAB1*+ subtype and fibroblasts and pericytes could be mediated by PRSS3-F2R within the PARs signaling pathway (**Figures 5J, K**).

### Integrating single-cell and ST to analyze intercellular spatial interactions

**Figure 6:**
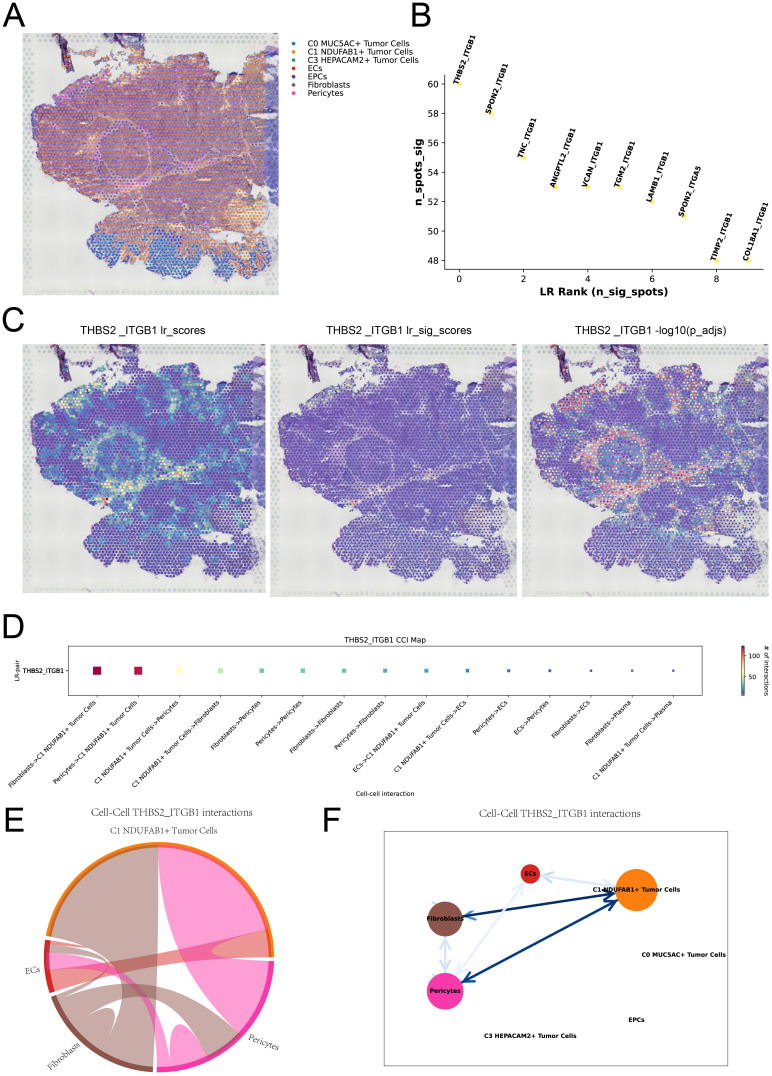
Spatial cell communication. **(A)** RCTD analysis predicted the cell types at each spatial spot on ST 2 slide. **(B)** The ranking plot displayed the top 10 significant ligand-receptor pairs within the spots. **(C)** The interaction strength of the THBS2-ITGB1 ligand-receptor pair was represented across all spots (left), in significant spots (middle), and its statistical value was shown for each spot (right). **(D)** The interaction heatmap visualized the intensity of intercellular interactions mediated by the THBS2-ITGB1 ligand-receptor pair. **(E, F)** Chord plot and network diagram depicted the spatial interactions among different cell types in the THBS2-ITGB1 ligand-receptor pair.

To further understand the spatial distribution and spatial interactions of different cell types within the TME, we integrated scRNA-seq data with ST data using the RCTD deconvolution technique, delineating the spatial architecture of different cell types on ST 2 slide (**Figure 6A**). Subsequently, we analyzed ST 2 slide using the “Stlearn” package in Python, displaying the top 10 significant ligand-receptor pairs within the spots in **Figure 6B**. We illustrated the spatial interaction strength and statistical value of the THBS2-ITGB1 interaction pair and performed spatial enrichment analysis. By combining the results with **Figure 6A**, we observed that the THBS2-ITGB1 interactions were predominantly concentrated at the boundaries of tumor cells. This suggested that the ligand-receptor interactions between THBS2 and ITGB1 might play a regulatory role in spatial communication between tumor cells and other cell types (**Figure 6C**). In **Figure 6D**, we visualized the strength of cell-cell interactions mediated by the THBS2-ITGB1 ligand-receptor pair, revealing higher communication intensity between C1 *NDUFAB1*+ subtype and fibroblasts as well as pericytes. The results in **Figures 6E, F** further confirmed this observation and unveiled the complexity of this spatial cell-cell communication pattern. Based on these findings, we speculated that C1 *NDUFAB1*+ subtype might have reshaped the network of intercellular interactions within the TME through abnormal communication crosstalk with fibroblasts and pericytes.

### Investigation of C1 *NDUFA*B1+ subtype based on TF regulatory networks

**Figure 7:**
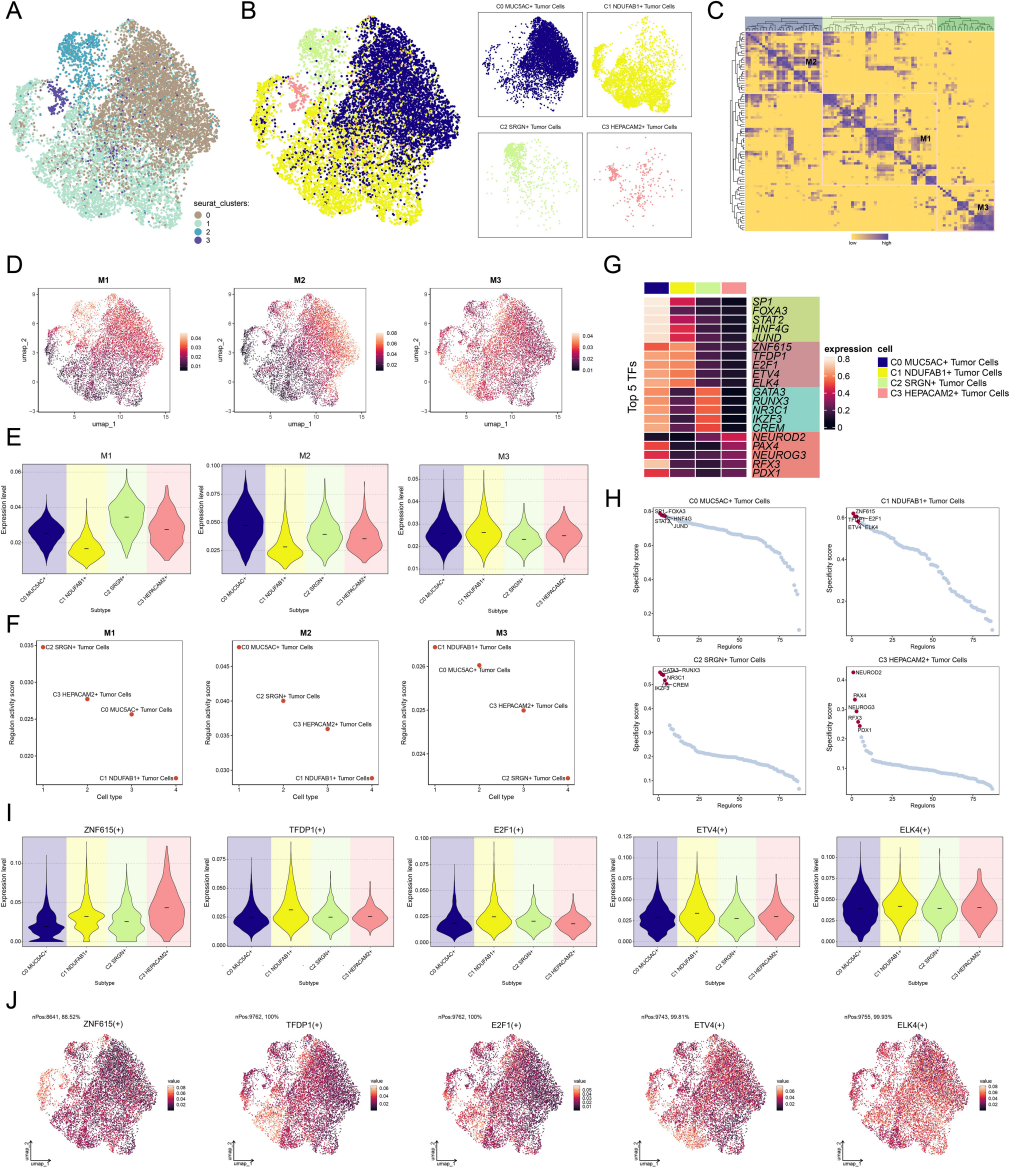
Characterization of TF activity and regulatory modules in tumor cell subtypes. **(A)** UMAP plot revealed the clustering patterns of tumor cells, which were determined by gene expression levels. **(B)** UMAP plots colored and visualized distinct clustering patterns among tumor cells based on the regulon activity scores. **(C)** Heatmap demonstrated the identification of three regulatory modules through hierarchical clustering of TFs within tumor cell subtypes. **(D)** UMAP plots presented the distribution of expression levels of TFs in each module. **(E)** Violin plots illustrated the expression levels of four tumor cell subtypes within each module. **(F)** Scatter plots demonstrated the ranking of regulon activity scores of TFs across four tumor cell subtypes within each module. **(G)** Heatmap exhibited the expression of the top 5 TFs in each tumor cell subtype. **(H)** Scatter plots demonstrated the ranking of specificity scores for the top 5 TFs in each tumor cell subtype. **(I)** Violin plots illustrated the expression levels of the top 5 TFs in C1 *NDUFAB1*+ subtype across each tumor cell subtype. **(J)** UMAP plots visualized the expression distribution of the top 5 TFs in C1 *NDUFAB1*+ subtype within the M3 module.

Investigating TFs is crucial for exploring tumor heterogeneity and the TME, as TFs play a pivotal role in regulating gene expression and cellular behavior. **Figure 7A** displayed the clustering analysis of tumor cells based on their gene expression levels, followed by the clustering patterns of different tumor cell subtypes according to regulon activity scores (**Figure 7B**). Subsequently, we identified three regulatory modules through hierarchical clustering of TFs within the tumor cell subtypes (**Figure 7C**). We visualized the expression distribution of TFs across each module and the expression levels of different tumor cell subtypes within each module (**Figures 7D, E**), revealing that C1 *NDUFAB1*+ subtype displayed elevated expression within the M3 module relative to other subtypes. Meanwhile, within the M3 module, C1 *NDUFAB1*+ subtype showed higher regulon activity scores relative to other tumor cell subtypes (**Figure 7F**). **Figure 7G** illustrated the top 5 TFs in C1 *NDUFAB1*+ subtype, namely ZNF615, TFDP1, E2F1, ETV4, and ELK4. In **Figure 7H**, we observed the top 5 TFs with higher specificity scores in C1 *NDUFAB1*+ subtype. We further analyzed the expression levels of the top 5 TFs in C1 *NDUFAB1*+ subtype across different subtypes (**Figure 7I**) and visualized their expression distribution within the M3 module using UMAP plots (**Figure 7J**). We noted that ELK4 exhibited elevated expression levels relative to other subtypes, and its expression within the M3 module was more pronounced relative to the other four TFs. Therefore, we conducted *in vitro* experiments to further investigate the effects of ELK4 on GC cells.

### *In vitro* experiments validated the regulatory role of ELK4 throughout the advancement of GC

**Figure 8:**
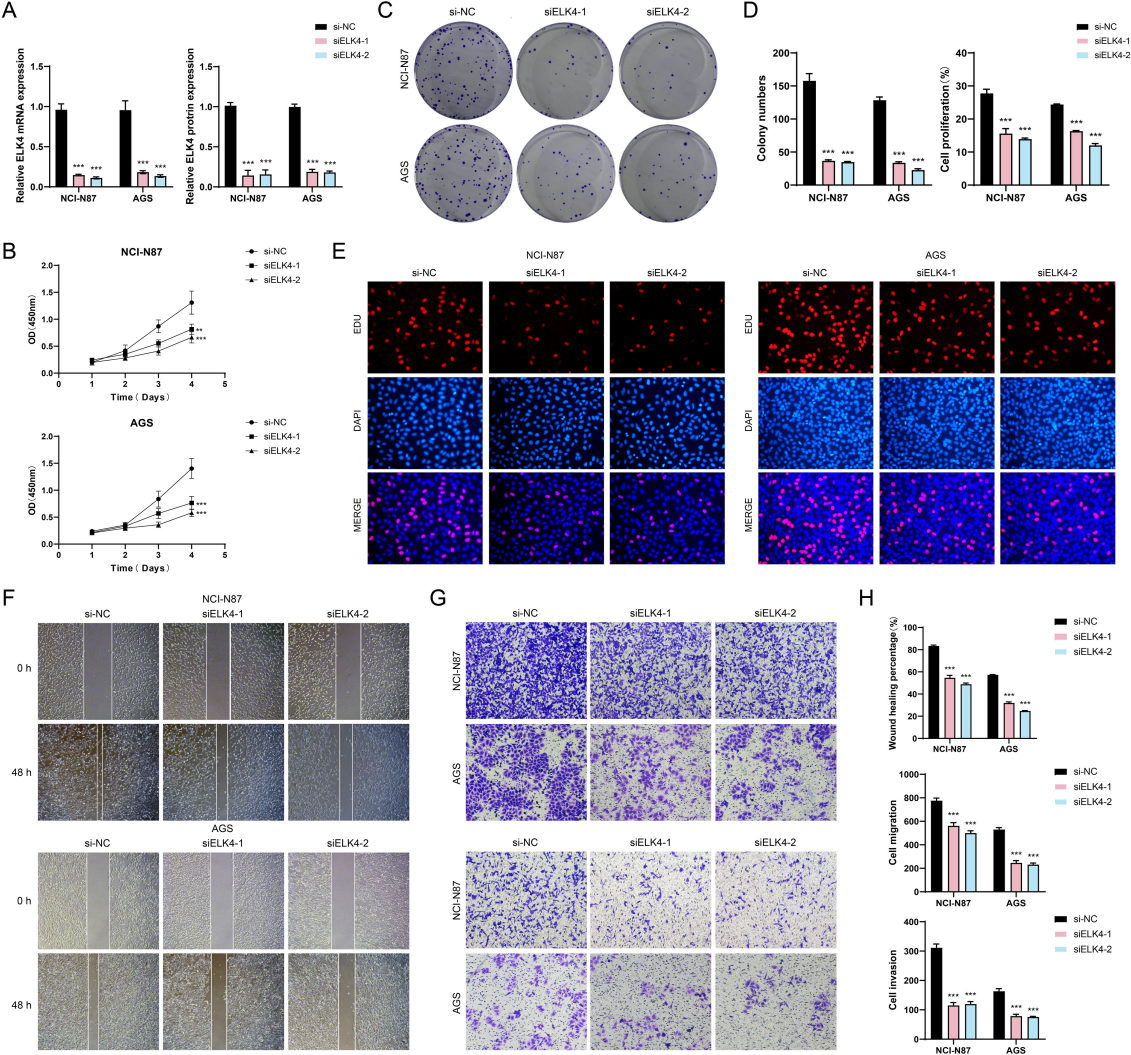
*In vitro* experimental validation. **(A)** Bar plots displayed the relative expression levels of ELK4 mRNA and ELK4 protein in the NCI-N87 and AGS GC cell lines for si-NC, siELK4-1, and siELK4-2. **(B)** The CCK-8 assay demonstrated the OD values of si-NC, siELK4-1, and siELK4–2 in the two GC cell lines. **(C)** The colony formation assays compared the results of cell colony formation among si-NC, siELK4-1, and siELK4–2 in the two GC cell lines. **(D)** Bar plots visually showed the colony numbers and cell proliferation rates of si-NC, siELK4-1, and siELK4–2 in the two GC cell lines. **(E)** The EDU staining assays exhibited the results of cell proliferation among si-NC, siELK4-1, and siELK4–2 in the two GC cell lines. **(F)** The cell wound healing assays evaluated the ability of cell wound healing at 0 h and 48 h among si-NC, siELK4-1, and siELK4–2 in the two GC cell lines. **(G)** Transwell assays evaluated the cell migration and invasion abilities among si-NC, siELK4-1, and siELK4–2 in the two GC cell lines. **(H)** Bar plots compared the results of cell wound healing, migration, and invasion among si-NC, siELK4-1, and siELK4–2 in the two GC cell lines. **P < 0.01, ***P < 0.001.

To gain deeper insights into the involvement of ELK4 in GC advancement, *in vitro* assay was carried out with NCI-N87 and AGS cell populations. Originally, we performed knockdown of ELK4 and then categorized into three distinct groups: si-NC, siELK4-1, and siELK4-2. Subsequently, we observed that the relative expression levels of both mRNA and protein were markedly decreased in the siELK4–1 and siELK4–2 groups when measured against the si-NC group (**Figure 8A**). Additionally, using the CCK-8 assay, we observed that the mean optical density (OD) values were also significantly decreased in the siELK4–1 and siELK4–2 groups (**Figure 8B**). This demonstrated that the cell viability of the NCI-N87 and AGS GC cell lines was significantly reduced after ELK4 knockdown. Next, colony formation and EDU staining results revealed that the colony numbers and the cell proliferation rates were markedly decreased in both GC cell populations after ELK4 knockdown (**Figures 8C–E**). Finally, the cell wound healing and transwell assays showed that the wound healing ability, migration, and invasion capabilities of the two GC cell lines were inhibited following ELK4 knockdown (**Figures 8F–H**). Through these studies, we uncovered ELK4’s crucial involvement in GC development, providing important experimental evidence for further exploration of ELK4 as a promising target for GC treatment.

### Construction and validation of a risk scoring model for tumor prognosis prediction

**Figure 9:**
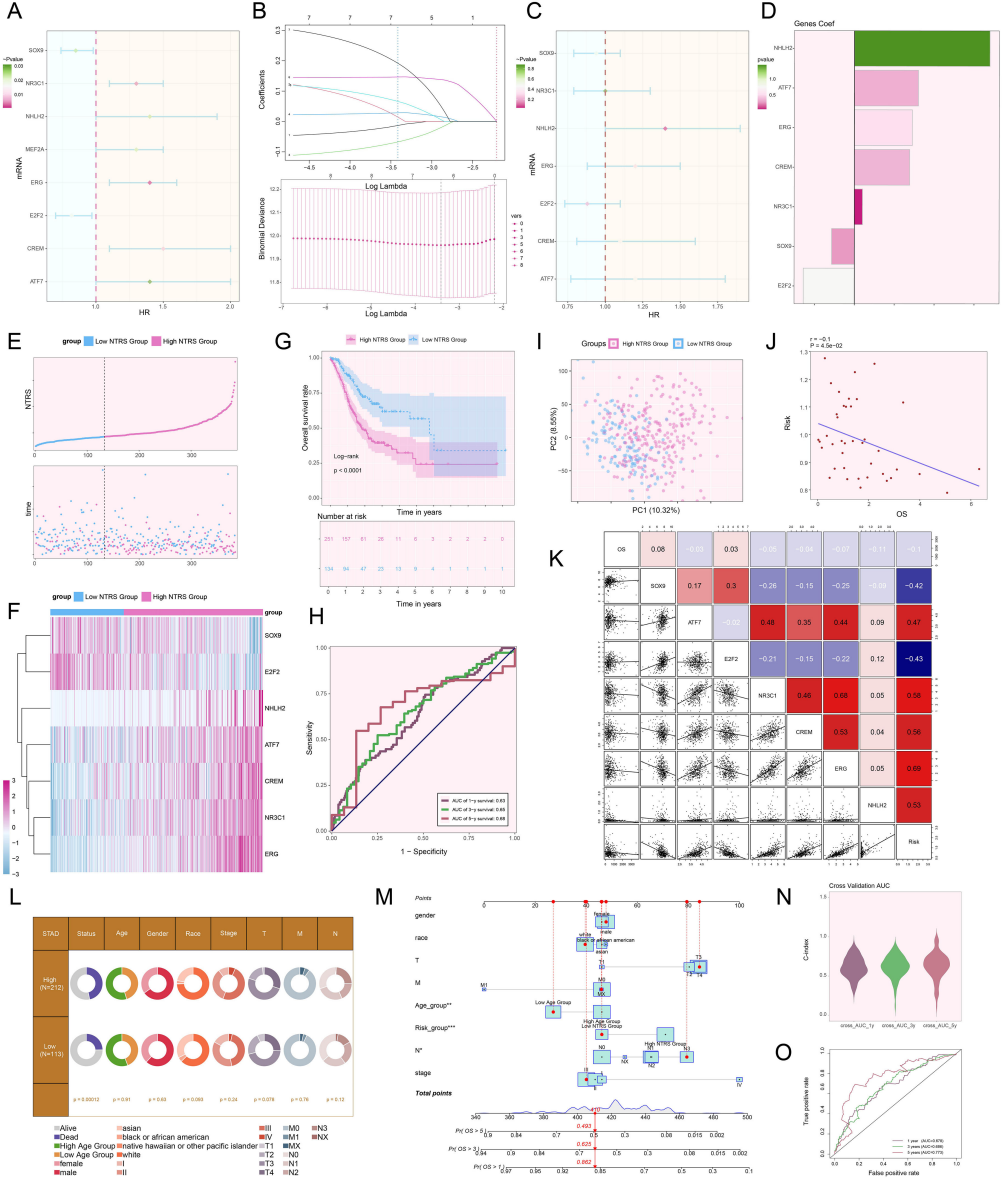
Creation and validation of a prognostic risk scoring model. **(A)** Forest plot demonstrated the results of univariate Cox regression analysis, where HR < 1 represented protective factors and HR > 1 indicated risk factors. **(B)** LASSO regression analysis identified prognostic genes by determining the optimal parameters through cross-validation (upper) and generating the coefficient profile based on the optimal lambda (lower). **(C)** Forest plot illustrated the results of multivariate Cox regression analysis. **(D)** Bar plot exhibited the coefficients of the genes in the constructed model. **(E)** The risk curve and scatter plot contrasted the risk scores (upper) and survival outcomes (lower) over time in the high and low NTRS groups, respectively. **(F)** Heatmap compared the differences in expression levels of prognostic genes in the constructed model between the high and low NTRS groups. **(G)** Kaplan-Meier curves compared the OS rates between the high and low NTRS groups. **(H)** ROC curves analysis for survival prediction showed sensitivity and specificity for 1-year, 3-year, and 5-year survival. **(I)** Scatter plot utilized PCA to demonstrate the differences in gene distribution between the high and low NTRS groups. **(J)** Scatter plot combined with a linear regression line showed the relationship between risk scores and OS. **(K)** Heatmap and scatter plots for correlation analysis, showing the correlation coefficients among prognostic genes, OS, and risk. **(L)** Pie chart compared the proportional distribution of clinical characteristics, including status, age, gender, race, stage, and TNM classification, between the high and low NTRS groups. **(M)** Nomogram predicted 1-year, 3-year, and 5-year OS using various clinical characteristics, including age, gender, race, stage, risk score, and TNM classification. *P < 0.05, **P < 0.01, ***P < 0.001. **(N)** Violin plot demonstrated the C-index for 1-year, 3-year, and 5-year predictions, measured by AUC through cross-validation. **(O)** ROC curves illustrated the predictive performance of the model through AUC values for 1-year, 3-year, and 5-year predictions.

We designed a risk scoring model to probe the molecular mechanisms of tumor prognosis and comprehensively assess patients’ prognostic risks. Initially, eight genes with notable prognostic relevance were ascertained (**Figure 9A**). To mitigate multicollinearity, narrowing down the selection to seven key prognostic genes (**Figure 9B**). In-depth profiling of these genes and their coefficients indicated that *NHLH2*, *ATF7*, *ERG*, *CREM*, and *NR3C1* were associated with poor prognosis (**Figures 9C, D**). Using the optimal threshold for the *NDUFAB1*+ tumor cells risk score (NTRS), patients were stratified into high and low NTRS groups, followed by DEGs analysis. The findings indicated notable disparities in the expression of the seven genes between the two groups, with the high NTRS group exhibiting poorer prognosis (**Figures 9E, F**). Kaplan-Meier survival curves further validated these findings, demonstrating lower OS rates in the high NTRS group (**Figure 9G**). To evaluate the model’s predictive performance, ROC curves were plotted. The AUC values for 1-year, 3-year, and 5-year survival predictions all exceeded 0.6, indicating the model’s robust predictive capability (**Figure 9H**). PCA demonstrated notable variations in gene distribution between the two groups, with PC1 and PC2 explaining 10.32% and 8.55% of the total variance, respectively (**Figure 9I**). Additionally, a negative correlation between risk scores and OS was observed, further supporting the model’s reliability (**Figure 9J**). Further analysis of the relationships among the seven prognostic genes, risk scores, and OS showed that *NHLH2*, *ATF7*, *ERG*, *CREM*, and *NR3C1* exhibited a positive correlation with risk scores but an inverse correlation with OS, suggesting their potential roles as drivers of poor prognosis. In contrast, *SOX9* and *E2F2* exhibited the opposite trends, indicating potential protective effects on prognosis (**Figure 9K**). To explore the influence of various risk factors on prognosis, the distribution of clinical features was compared between the high and low NTRS groups (**Figure 9L**). A nomogram incorporating age, gender, race, stage, risk score, and TNM classification was constructed, demonstrating that the NTRS risk group exhibited the most significant survival differences (**Figure 9M**). Finally, the model’s accuracy was validated using the C-index and true positive rate, with AUC values for 1-year, 3-year, and 5-year predictions all exceeding 0.6, further confirming the model’s robustness (**Figures 9N, O**). These findings provide critical theoretical and practical insights for optimizing clinical decision-making, accurately predicting patient prognosis, and improving survival outcomes.

### Investigating the effect of NTRS on the immune landscape and prognosis in tumor cells

**Figure 10:**
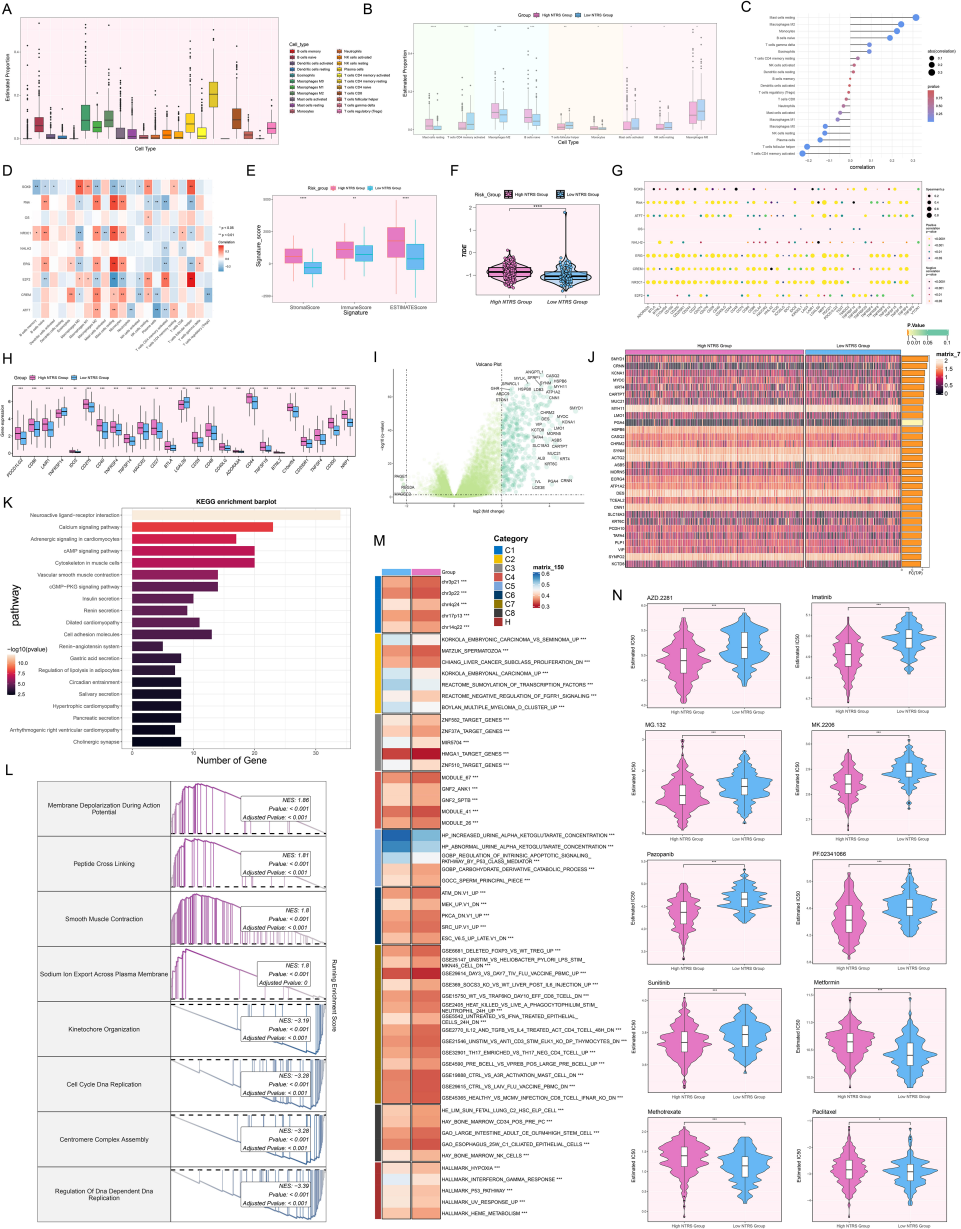
Analysis of immune profiling, enrichment, and drug sensitivity in the prognostic model. **(A)** Box plot displayed the estimated proportions of 22 immune cell types. **(B)** Box plot showcased significant differences in the estimated proportions of nine immune cell types between the high and low NTRS groups. **(C)** Lollipop plot revealed the results of the correlation analysis between different immune cell types and the risk score. **(D)** Heatmap manifested the correlation between different immune cell types and prognostic genes, OS, and risk scores. **(E)** Box plot compared the differences in signature scores (stromal, immune, and ESTIMATE) between the high and low NTRS groups. **(F)** Violin plot exhibited the comparison of TIDE score between the high and low NTRS groups. **(G)** Bubble plot presented the results of Spearman correlation analysis between different immune checkpoint-related genes and prognostic genes, OS, and risk scores. **(H)** Box plot showed significant differences in the expression of various immune checkpoint-related genes between the high and low NTRS groups. **(I)** Volcano plot exhibited the DEGs between the high and low NTRS groups. **(J)** Heatmap revealed significant differences in gene expression between the high and low NTRS groups. **(K)** Bar plot displayed the top 20 enriched pathways of DEGs in the KEGG enrichment analysis. **(L)** GSEA enrichment analysis of DEGs revealed the enrichment results of major biological processes. **(M)** GSVA enrichment analysis showed the main biological pathways and gene sets enriched in the high and low NTRS groups. **(N)** Violin plots combined with box plots illustrated the drug sensitivity differences between the high and low NTRS groups by comparing the IC50 values assessed using different therapeutic drugs. *P < 0.05, **P < 0.01, ***P < 0.001, and ****P < 0.0001.

The initial step in examining NTRS’s complex involvement in the immune microenvironment involved determining the relative abundance of distinct immune cell populations (**Figure 10A**). **Figure 10B** manifested pronounced contrasts regarding the estimated proportions of nine immune cell types between both groups. In the high NTRS group, MCs resting, Macrophages M2, B cells naive, and Monocytes were more abundant. In contrast, T cells CD4 memory activated, T cells follicular helper, MCs activated, NK cells resting, and Macrophages M0 were more prevalent in the low NTRS group. Comprehensive analyses were performed to evaluate the interconnections among diverse immune cell types and risk scores, prognostic genes, and OS (**Figures 10C, D**). MCs resting, Macrophages M2, Monocytes, and B cells naive showed significant positive correlations with risk scores. Conversely, T cells CD4 memory activated and T cells follicular helper exhibited significant negative correlations with risk scores. These findings suggested that the observed differences in immune cell composition across study groups might be closely related to disease risk and prognosis. Next, we compared the differences in signature and TIDE scores between the high and low NTRS groups (**Figures 10E, F**). The results indicated that the stromal, immune, ESTIMATE, and TIDE scores were significantly higher in the high NTRS group, suggesting a more immunosuppressive TME in this group. Additionally, analysis outcomes of immunological checkpoint-associated genes (**Figure 10G**) uncovered that the majority immune checkpoints were positively correlated with risk scores. Genes such as *ATF7*, *ERG*, *CREM*, *NR3C1*, and *E2F2* were positively correlated with multiple immune checkpoints, while *NHLH2* and *SOX9* showed negative correlations. In the high NTRS group, most immune checkpoint-related genes were expressed at higher levels, with only *TNFRS14* and *LGALS9* showing higher expression in the low NTRS group (**Figure 10H**).

To comprehensively reveal the discrepancies between both groups, we probed the upregulated and downregulated DEGs and distinct gene expression patterns (**Figures 10I, J**). DEGs were found to be predominantly enriched in pathways such as neuroactive ligand-receptor interaction, calcium signaling pathway, adrenergic signaling in cardiomyocytes, cAMP signaling pathway, and cytoskeleton in muscle cells (**Figure 10K**). GSEA enrichment analysis (**Figure 10L**) further demonstrated that upregulated genes were primarily engaged in biological processes such as membrane depolarization during action potential, peptide cross linking, smooth muscle contraction, and sodium ion export across plasma membrane. Downregulated genes were chiefly implicated in kinetochore organization, cell cycle DNA replication, and centromere complex assembly. GSVA analysis highlighted significant differences in major biological pathways and gene set enrichment between both groups (**Figure 10M**). Finally, we evaluated the sensitivity to different therapeutic drugs (**Figure 10N**). The high NTRS group showed greater sensitivity to AZD.2281, Imatinib, MG.132, MK.2206, Pazopanib, PF.02341066, and Sunitinib. In contrast, the low NTRS group exhibited higher sensitivity to Metformin, Methotrexate, and Paclitaxel. These findings not only revealed the complex regulatory network of NTRS within the immune landscape and its possible influence on disease prognosis but also laid a crucial foundation for developing precise treatment strategies tailored to different risk score groups.

## Discussion

GC maintains a notable position in the epidemiology of digestive system cancers (88). Owing to nonspecific initial clinical manifestations, most cases are diagnosed during progressive disease phases, leading to unfavorable outcomes and a 5-year OS rate below 5% (89). Although the treatment options for GC are diverse, such as surgical removal, chemotherapy, radiation therapy, targeted treatments, and immunotherapeutic approaches, these methods still face significant limitations in practical applications. Firstly, tumor heterogeneity leads to substantial differences in treatment responses among distinct patients or even within different regions of the same tumor, greatly limiting the universality and effectiveness of treatments (90). Secondly, drug resistance is a particularly prominent issue (91), with many patients developing resistance after initial treatment, leading to disease progression (92). Additionally, the complexity of the TME further exacerbates treatment challenges, as the immunosuppressive cells within, such as tumor-associated macrophages and regulatory T cells, promote immune evasion and drug resistance in tumor cells by secreting immunosuppressive factors and metabolites (93–95). Meanwhile, GC cells adapt to microenvironmental changes through metabolic reprogramming, such as enhanced glycolysis, glutamine metabolism, and fatty acid synthesis, to maintain their survival advantages, which also provides a potential mechanism for treatment resistance (96). These factors not only limit the effectiveness of current treatments but also highlight the urgent need to develop new therapeutic strategies.

ScRNA-seq technology not only reveals the molecular characteristics of GC but also deciphers makeup and operational dynamics of various cellular entities in the TME, offering essential perspectives on tumor heterogeneity, drug resistance mechanisms, and immune evasion (97–99). In this research, we pinpointed 10 key cell types that were pivotal in the advancement of GC. Notably, the heterogeneity of EPCs, particularly the identification of four distinct tumor cell subtypes (C0 *MUC5AC*+, C1 *NDUFAB1*+, C2 *SRGN*+, and C3 *HEPACAM2*+), highlighted the complexity of GC biology. Among these tumor cells, C1 *NDUFAB1*+ subtype demonstrated elevated expression of genes associated with the cell cycle and indicators of cellular activity, suggesting a more aggressive phenotype. This subtype was more prevalent in intestinal-type GC samples, aligning with its potential role in driving tumor progression. We also observed that C1 *NDUFAB1*+ subtype showed a preference for the G2/M and S phases, further supporting their proliferative and potentially more malignant characteristics. ST analysis further confirmed these findings, revealing the spatial enrichment of C1 *NDUFAB1*+ subtype in specific regions of GC tissues. These findings not only supported the proliferative activity and malignant features of C1 *NDUFAB1*+ subtype but also revealed their spatial heterogeneity within the TME. The specific spatial localization patterns of C1 *NDUFAB1*+ subtype in tumor tissues were likely closely related to their functional role in tumor progression.

The investigation additionally uncovered notable distinctions in the biological process and metabolic profiles among each subtype. The results showed that C1 *NDUFAB1*+ subtype was primarily associated with mitochondrial function, localization, and triphosphate metabolism, and was significantly enriched in biological processes including cytoplasmic translation, oxidative phosphorylation, aerobic respiration, ATP synthesis coupled electron transport, and mitochondrial ATP synthesis coupled electron transport. These findings indicated that C1 *NDUFAB1*+ subtype exhibited high metabolic activity, which likely provided energy support for their rapid proliferation and survival, further solidifying their role as a critical driving subtype in tumor progression. Additionally, C1 *NDUFAB1*+ subtype also demonstrated significant activity in metabolic pathways encompassing oxidative phosphorylation, glutathione metabolism, glycolysis/gluconeogenesis, sulfur metabolism, and citrate cycle (TCA cycle), further emphasizing their critical role in tumor metabolic reprogramming (100, 101). In contrast, other tumor cell subtypes exhibited distinct functional characteristics. C0 *MUC5AC*+ subtype was mainly associated with cell junctions, RNA splicing, and miRNA metabolism, potentially playing a role in maintaining intercellular communication and transcriptional regulation (102). C2 *SRGN*+ subtype was significantly enriched in pathways related to leukocyte-mediated immune responses and the regulation of cell-cell adhesion, suggesting their potential role in the tumor immune microenvironment (103, 104). Meanwhile, C3 *HEPACAM2*+ subtype was primarily linked to protein folding and endoplasmic reticulum stress responses, possibly functioning in cellular stress responses and the maintenance of protein homeostasis (105). These findings highlighted the functional heterogeneity of GC tumor cell subtypes and revealed the diverse roles of different subtypes in tumor progression.

The differentiation trajectories and stemness analysis of tumor cell subtypes in GC provided profound insights into the cellular dynamics and functional heterogeneity within the TME. Our findings revealed that C1 *NDUFAB1*+ subtype displayed greater stem-like characteristics relative to other subtypes, suggesting that these cells might possess stronger self-renewal and differentiation potential (106). Stemness genes were essential for supporting the self-renewal, preservation, and differentiation potential of cancer stem cells (107, 108). The high stemness of C1 *NDUFAB1*+ subtype was likely supported by the elevated expression of key stemness genes. *CTNNB1*, a pivotal molecule in the Wnt signaling pathway, was involved in cell proliferation and stemness maintenance (109). *MYC*, a proto-oncogene, regulated the cell cycle and metabolism (110, 111). *ABCG2*, a multidrug resistance protein, was potentially associated with treatment resistance in tumor cells (112, 113). *LGR5*, a target gene of the Wnt signaling pathway, was commonly used to mark tumor stem cells (114). The high stemness and metabolic activity of C1 *NDUFAB1*+ subtype suggested that they might play a critical role in driving tumor advancement and resistance to therapy (115). The differentiation trajectories inferred through Slingshot analysis further highlighted the influential role of C1 *NDUFAB1*+ subtype in the developmental dynamics of GC cells. Both lineage 1 and lineage 2 originated from C0 *MUC5AC*+ subtype, which exhibited lower stemness and higher differentiation levels, and diverged after passing through C1 *NDUFAB1*+ subtype. This observation suggested that C1 *NDUFAB1*+ subtype might act as a transitional or progenitor-like population, driving the differentiation of tumor cells into distinct functional states. The pseudotime trajectories of named genes within these lineages further validated this hypothesis, showing a clear progression along the inferred differentiation pathways. Functional enrichment analysis of the differentiation lineages revealed cluster-specific biological processes. For instance, the C1 cluster was linked to immune and humoral responses, suggesting a potential role in modulating the tumor immune microenvironment. The C2 cluster showed a connection to digestive and structural functions, possibly reflecting its involvement in maintaining tissue architecture and function. The C3 cluster showed a strong connection to the Wnt signaling pathway (116) and morphogenesis, indicating its potential effect on regulating cell fate and tissue patterning. Finally, the C4 cluster was related to visual, brain, and pancreatic functions, hinting at a broader role in systemic tumor biology. These findings underscored the functional diversity and complexity of tumor cell subtypes in GC. The high stemness and differentiation potential of C1 *NDUFAB1*+ subtype, coupled with their central role in differentiation trajectories, suggested that this subtype might function as an important driver of tumor progression and heterogeneity.

Next, we carried out a detailed exploration of the cell-cell communication network of C1 *NDUFAB1*+ subtype within the TME using CellChat. The results revealed that C1 *NDUFAB1*+ subtype communicated with fibroblasts and pericytes through the PARs signaling pathway. Proteinase activated-receptors (PARs), a class of G protein-coupled receptors, have been demonstrated to play important roles in various cancers (117–119). Particularly in GC, the expression of PARs was closely associated with tumor development and progression (120, 121). The role of the PARs signaling pathway in the TME has been extensively studied, especially in the interactions between tumor cells and stromal cells (122, 123). We observed that PRSS3 and F2R were highly expressed in C1 *NDUFAB1*+ subtype, fibroblasts, and pericytes, suggesting that the PRSS3-F2R ligand-receptor pair might contribute significantly to mediate communication between these cells. This discovery provided new insights into the molecular mechanisms of cell-cell communication in the TME. To achieve a deeper insight into the spatial distribution and interactions of different cell types within the TME, we integrated scRNA-seq data with ST data. We found that the THBS2-ITGB1 ligand-receptor pair exhibited particularly significant interactions at the boundaries of tumor cells. This phenomenon suggested that THBS2-ITGB1 might act as an important regulator in spatial communication between tumor cells and other cell types. By visualizing the strength of cell-cell interactions facilitated by THBS2-ITGB1, we observed higher communication intensity between C1 *NDUFAB1*+ subtype and fibroblasts, as well as pericytes. This result further supported the hypothesis that C1 *NDUFAB1*+ subtype reshaped the network of intercellular interactions within the TME through abnormal signaling crosstalk. Such aberrant communication patterns may have provided favorable conditions for tumor multiplication, infiltration, and metastasis. Through integrating spatial transcriptomic data with histomorphological information, we not only revealed the spatial distribution characteristics of the C1 *NDUFAB1*+ subtype but also provided a more comprehensive interpretation of its biological significance in tumor progression.

In this study, we conducted a meticulous exploration of C1 *NDUFAB1*+ subtype through TF regulatory networks. TFs orchestrate the regulation of gene expression and cellular behavior (124, 125), making their investigation crucial for understanding tumor heterogeneity and the TME. Further research revealed that C1 *NDUFAB1*+ subtype exhibited higher expression levels and regulon activity scores in the M3 module, suggesting that this module might have been a significant player in the functional regulation of C1 *NDUFAB1*+ subtype. Within the M3 module, C1 *NDUFAB1*+ subtype displayed higher expression of specific TFs, particularly the top 5 TFs: ZNF615, TFDP1, E2F1, ETV4, and ELK4. Among these, the expression level of ELK4 was particularly prominent in C1 *NDUFAB1*+ subtype, significantly higher than in other tumor cell subtypes. This finding suggested that ELK4 potentially served as a critical factor in the biological behavior of C1 *NDUFAB1*+ subtype. Prior studies demonstrated that ELK4, an ETS-family TF, promoted GC progression by activating oncogenic lncRNA SNHG22, and its knockdown suppressed GC cell proliferation and invasion (126). Additionally, ELK4 was shown to drive malignant phenotypes in GC by regulating the KDM5A-PJA2-KSR1 axis (127). To validate this hypothesis, we further investigated the effects of ELK4 on the proliferation, migration, and invasion capabilities of GC cells through *in vitro* functional experiments.

*In vitro* studies indicated that silencing ELK4 markedly hindered the growth of NCI-N87 and AGS cell strains. Using CCK-8 assays, colony formation assays, and EDU staining, we observed that the viability and proliferation rates of GC cells considerably diminished after ELK4 knockdown. Additionally, wound healing and transwell assays further confirmed that the migration and invasion capabilities of GC cells were markedly suppressed following ELK4 knockdown. These results collectively implied that ELK4 served as a critical factor in modulating the oncogenic properties of GC cells.

By integrating multi-omics data and clinical prognostic information, we designed and confirmed a predictive risk scoring model using C1 *NDUFAB1*+ subtype. The results demonstrated that *NHLH2*, *ATF7*, *ERG*, *CREM*, and *NR3C1* were associated with poor prognosis, while *SOX9* and *E2F2* exhibited potential protective effects. Using the optimal threshold for NTRS, patients were grouped into high and low NTRS cohorts, where the high NTRS cohort showed significantly poorer survival rates. We thoroughly investigated the impact of C1 *NDUFAB1*+ subtype on the immune microenvironment, particularly their roles in immune escape and metabolic reprogramming. In the high NTRS group, the proportions of MCs resting, Macrophages M2, B cells naive, and Monocytes were higher, which are typically associated with an immunosuppressive microenvironment (128–131). In contrast, the low NTRS group exhibited higher proportions of T cells CD4 memory activated, T cells follicular helper, MCs activated, NK cells resting, and Macrophages M0, which are generally linked to an immune-activated state (132–135). These differences in immune cell distribution may have reflected the ability of the high NTRS group to evade immune surveillance through immune escape mechanisms, thereby promoting tumor progression. Further analysis revealed that MCs resting, Macrophages M2, Monocytes, and B cells naive were significantly positively correlated with the risk score, while T cells CD4 memory activated and T cells follicular helper were negatively correlated with the risk score. These evidences reflected that the TME in the high NTRS group might be more immunosuppressive, providing favorable conditions for tumor cell immune escape. Additionally, the high NTRS group showed significantly higher stromal, immune, ESTIMATE, and TIDE scores, further supporting the hypothesis of an immunosuppressive microenvironment in the high NTRS group. The elevated TIDE score, often associated with enhanced immune escape capabilities of tumor cells (136, 137), indicated that the high NTRS group might evade immune system attacks through multiple mechanisms. During this analysis, we observed a positive correlation between *SOX9* expression and patient prognosis, but a negative correlation with immune checkpoint markers. This suggested that the primary mechanism by which *SOX9* improved patient outcomes might be independent of or only weakly related to the immune system, instead operating through direct effects on tumor cell-intrinsic biological behaviors. Future studies should further investigate the precise molecular mechanisms through which *SOX9* regulates malignant phenotypes in GC cells and the immune microenvironment. These findings indicate that clinical practice should comprehensively consider both the tumor biological characteristics and immune microenvironment features reflected by *SOX9* expression levels to develop more precise treatment strategies.

KEGG and GSEA enrichment analyses revealed striking differences in metabolic pathways between both groups. The high NTRS group’s DEGs were primarily mapped to metabolic pathways such as neuroactive ligand-receptor interaction, calcium signaling pathways, and cAMP signaling pathways. The activation of these pathways might have promoted metabolic reprogramming in tumor cells (138–140), enabling them to adapt to nutrient deprivation and hypoxic conditions in the TME, thereby sustaining their proliferation and survival. Notably, these metabolic reprogramming features were highly consistent with the metabolic activity of the previously mentioned C1 *NDUFAB1*+ subtype. This metabolic reprogramming not only provided energy for tumor cells but also supplied precursor molecules for biosynthesis, supporting the maintenance of their malignant phenotype (141). Finally, we conducted extensive drug sensitivity screening and found that the high NTRS group was more sensitive to drugs such as AZD.2281, MG.132, and Pazopanib. These findings helped identify novel potential therapeutic targets, elucidate resistance mechanisms, and provide preliminary clues for future drug repositioning or combination therapy research.

This study revealed that the unique metabolic characteristics of the C1 *NDUFAB1*+ subtype not only supported its malignant phenotype but also potentially mediated treatment resistance. The concurrent activation of both oxidative phosphorylation and glycolysis pathways in this subtype enabled it to switch energy production modes in response to microenvironmental stresses, which likely represented a key mechanism underlying the failure of conventional chemotherapy and targeted therapies. Particularly noteworthy was the significant activation of the glutathione metabolic pathway, which might have further promoted drug resistance by scavenging reactive oxygen species and protecting tumor cells from chemotherapeutic damage (142, 143). The hyperactive TCA cycle and mitochondrial function in the C1 *NDUFAB1*+ subtype suggested its potential adaptive resistance to glycolysis-targeted therapies. Furthermore, this study revealed that high-NTRS patients exhibited significant immunosuppressive microenvironment characteristics, manifested by increased infiltration of immunosuppressive cells such as Macrophages M2 and MCs resting, accompanied by suppressed T cell function. This immunosuppressive state was closely associated with the unique metabolic reprogramming features of the C1 *NDUFAB1*+ subtype, indicating that targeting metabolic-immune interactions might represent a novel strategy to overcome immune evasion. Based on these findings, we proposed the following therapeutic strategies: First, targeting key metabolic reprogramming pathways (such as oxidative phosphorylation and glycolysis) and developing drugs targeting *NDUFAB1* or ELK4 specifically disrupted the metabolic hub of this subtype, effectively inhibiting its energy supply and consequently limiting its proliferation. Second, combined application of mitochondrial inhibitors with glycolysis blockers could help overcome monotherapy resistance caused by “metabolic compensation.” Additionally, combining ELK4 targeting with TME feature-based prognostic models, along with co-administration of immune checkpoint inhibitors and metabolic modulators, could effectively counteract immunosuppression, overcome immune evasion, and enhance anti-tumor immune responses. Finally, patients were classified by detecting characteristic metabolic markers of the C1 *NDUFAB1*+ subtype (including *NDUFAB1* and ELK4 expression levels as well as metabolic enzyme activity profiles), which guided personalized treatment selection. A risk scoring model incorporating metabolic-immune features (integrating parameters such as mitochondrial function indicators and immunosuppressive cell infiltration levels) was explored in combination with other emerging modalities (144–146) to predict patient responses to conventional chemotherapy and immunotherapy, as well as prognosis.

This study provided new targets and strategies for the precision treatment of GC, particularly demonstrating significant advantages in combining targeted metabolic pathways with immunomodulatory therapies. However, there are some limitations. First, the study was constrained by the sample size and geographical origin, which may not fully reflect the broad heterogeneity of GC, especially the differences across various molecular subtypes. Second, the existing ST data and resolution limitations (147) made it difficult to precisely analyze the interactions between individual tumor cells and specific stromal cells. Additionally, although *in vitro* experiments confirmed the regulatory role of ELK4, further *in vivo* studies were needed to validate its function in the intact TME. Due to the prolonged experimental timeline and complex sample acquisition procedures, the collection, processing, and staining analysis of samples could not be completed within the short term. Subsequent studies needed to establish prospective cohorts incorporating systematic staining analyses of both *NDUFAB1* and ELK4 into the research design. Finally, although we built a risk prediction framework, its clinical application still requires validation through large-scale prospective cohort studies to ensure its clinical utility and reliability. Future studies should further explore the molecular characteristics and clinical translational value of the C1 *NDUFAB1*+ subtype and its key regulator ELK4 in different GC subtypes, and deepen the understanding of the metabolic-immune interaction mechanisms through multi-omics integrated analysis.

## Conclusion

This study successfully deciphered the high heterogeneity of GC through scRNA-seq technology and identified the C1 *NDUFAB1*+ subtype as a critical cell population. In-depth analysis revealed that this subtype exhibited unique metabolic reprogramming characteristics, including significantly activated oxidative phosphorylation and glycolysis pathways, which potentially drove malignant tumor progression by providing energy and biosynthetic precursors. To further investigate the microenvironmental features of this subtype, we employed ST technology to elucidate its spatial distribution pattern in tumor tissues, discovering its enrichment at the tumor-stroma interface with observed strong spatial interactions. This distinct spatial distribution pattern not only enhanced the understanding of tumor-immune microenvironment interactions but also held potential clinical translational value, such as predicting tumor progression or therapeutic response based on spatial distribution features. *In vitro* experiments demonstrated that ELK4, as a key molecule of the C1 *NDUFAB1*+ subtype, significantly promoted the proliferation, migration, and invasion of GC cells. Based on these findings, we constructed a prognostic risk scoring model, which provided an important tool for patient stratification and personalized treatment. In summary, this study systematically elucidated the core mechanisms through which ELK4 mediated tumor progression, metabolic reprogramming, and immune evasion in C1 *NDUFAB1*+ subtype, offering crucial theoretical foundations for developing precision therapeutic strategies targeting this subtype.

## Data Availability

The original contributions presented in the study are included in the article/**Supplementary Material**. Further inquiries can be directed to the corresponding authors.

## Supplementary material

Supplementary Figure 1 Analysis of inferCNV. **(A, B)** Heatmaps compared the expression distribution of CNVs between ECs and EPCs (upper) and between ECs and four tumor cell subtypes (lower), where red represented amplification and orange represented deletion.

Supplementary Figure 2 Spatial expression pattern analysis of C1 *NDUFAB1*+ subtype. **(A, B)** The ST feature maps displayed the first cell types inferred at selected points on ST 2 slide and the spatial expression pattern of the C1 *NDUFAB1*+ subtype. **(C)** The ST feature maps visualized the spatial expression of nCount-Spatial, nFeature-Spatial, G2/M.Score, and S.Score for all cell types in ST 2 slide.
