# Supplementary figures and images for: Integrative single-cell and spatial transcriptomics uncover ELK4-mediated mechanisms in NDUFAB1+ tumor cells driving gastric cancer progression, metabolic reprogramming, and immune evasion
Source: Front Immunol. 2025 Jul 4;16:1591123. doi: 10.3389/fimmu.2025.1591123 (PMC12271198; doi:10.3389/fimmu.2025.1591123)

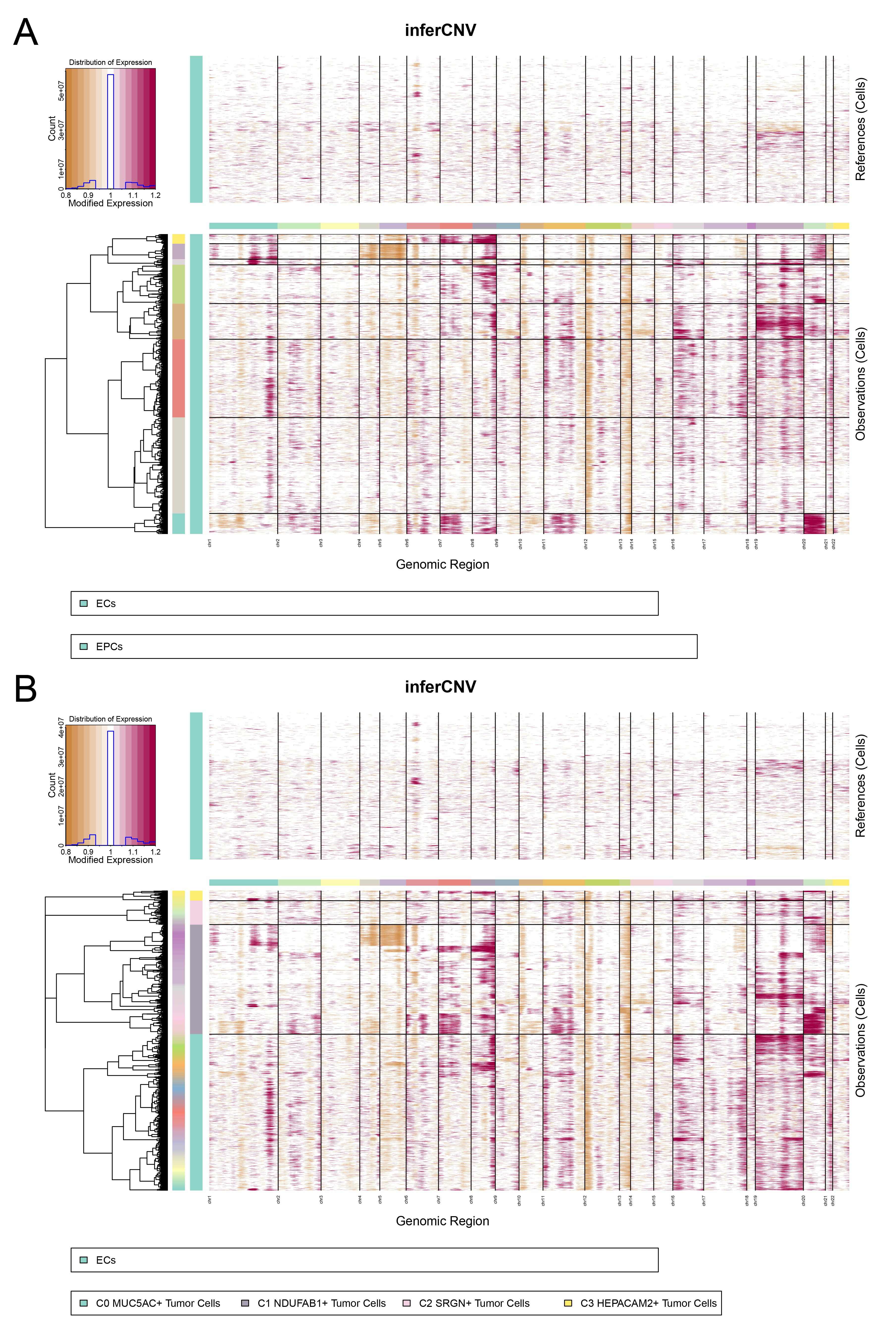

Supplement: Supplementary Figure 1 — Analysis of inferCNV. (A, B) Heatmaps compared the expression distribution of CNVs between ECs and EPCs (upper) and between ECs and four tumor cell subtypes (lower), where red represented amplification and orange represented deletion. [file Image1.jpeg]

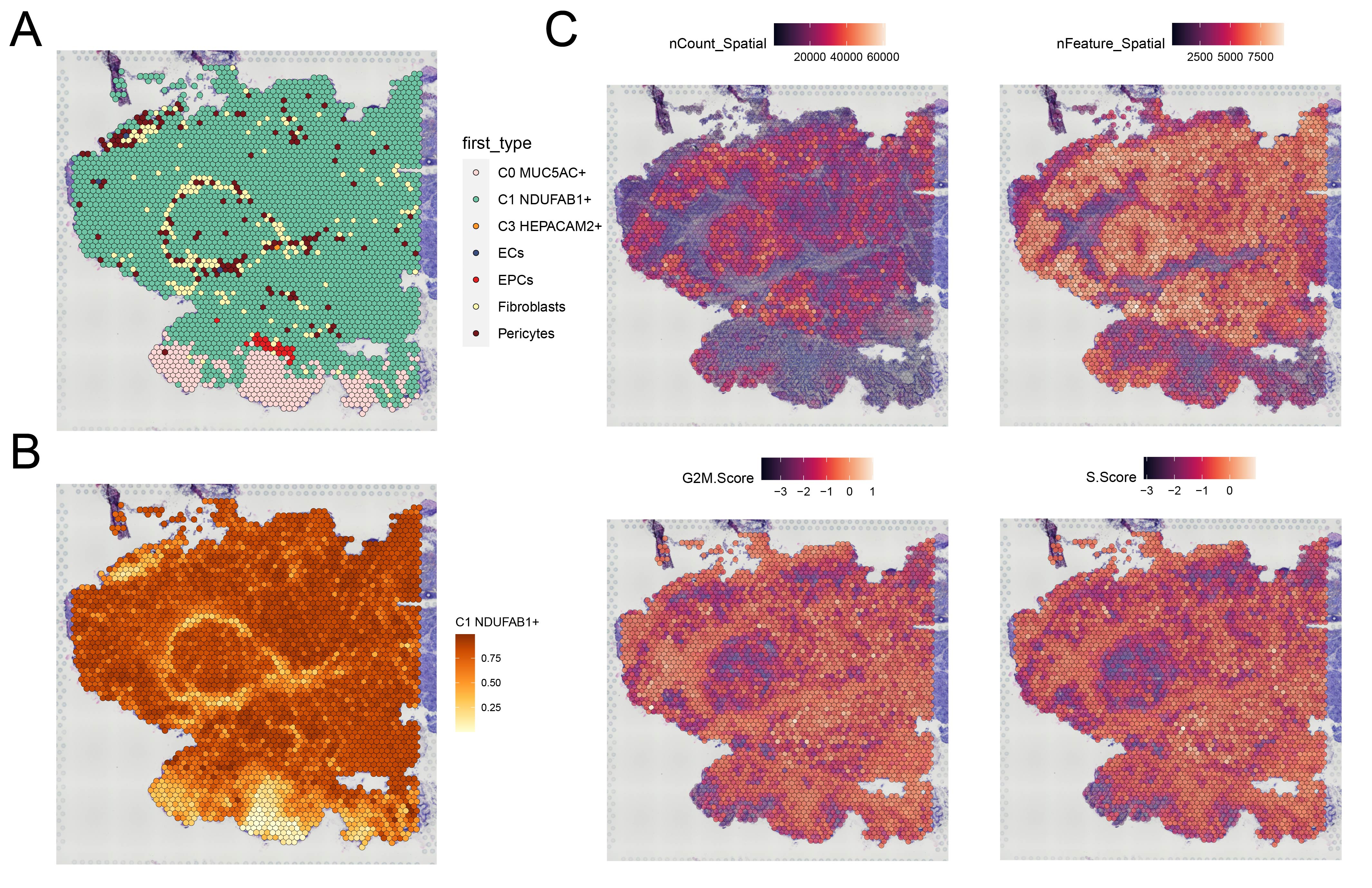

Supplement: Supplementary Figure 2 — Spatial expression pattern analysis of C1 NDUFAB1+ subtype. (A, B) The ST feature maps displayed the first cell types inferred at selected points on ST 2 slide and the spatial expression pattern of the C1 NDUFAB1+ subtype. (C) The ST feature maps visualized the spatial expression of nCount-Spatial, nFeature-Spatial, G2/M.Score, and S.Score for all cell types in ST 2 slide. [file Image2.jpeg]
